# Household-level food-insecurity prediction from experience-scale surveys and open geospatial data: A reproducible machine-learning pipeline for Nigeria

**DOI:** 10.1371/journal.pone.0349978

**Published:** 2026-10-07

**Authors:** Philip Osung Osung

**Affiliations:** Department of Economics, Nile University of Nigeria, Abuja, Nigeria; SKYDA Health Nigeria, NIGERIA

## Abstract

This paper develops a reproducible machine-learning pipeline for household-level food-insecurity prediction in Nigeria using the 2024 NDHS FIES module and five open geospatial layers. Because the NDHS is a two-stage cluster sample, all evaluation uses cluster-grouped data splits in which no enumeration area contributes households to both training and test sets. On a held-out test set of 7,999 households from 276 enumeration areas, the random forest reaches AUROC 0.727 (cluster-bootstrap 95 percent CI 0.704 to 0.747) and gradient-boosted trees 0.714 (0.690 to 0.737), against 0.679 (0.653 to 0.702) for a logistic baseline. Under leave-zone-out validation, which scores entire geopolitical zones the model never saw, the logistic baseline outperforms both ensembles in all six zones. SHAP attribution assigns between 42 and 52 percent of predictive signal to contextual predictors depending on model and coordinate treatment, a larger share than prior DHS tree-ensemble work reports. Travel time to the nearest city is associated with lower food-insecurity risk, a pattern consistent with a subsistence-buffer channel during sharp naira depreciation that we present as a hypothesis requiring replication.

## Introduction

Nigeria’s 2024 Demographic and Health Survey was the first round in the Nigerian DHS series to include the Food Insecurity Experience Scale module. It reported that 67.7 percent of the Nigerian de jure population lives in households experiencing moderate-or-severe food insecurity, and 33.0 percent experience severe food insecurity [1]. The 2021 Nigeria Multiple Indicator Cluster Survey (MICS) round also included a FIES module and has been used in published analyses of Nigerian food-insecurity prevalence and correlates. This paper is the first to apply machine-learning methods to a DHS-administered FIES outcome for Nigeria. The 2024 NDHS was fielded against a backdrop of significant macroeconomic shocks, including the June 2023 naira devaluation and the May 2023 removal of fuel subsidies, which together drove sharp increases in food-price inflation. The burden is spatially concentrated. Nasarawa records the lowest state-level prevalence at 37.9 percent. Yobe records the highest at 94.1 percent. Between these extremes sit Borno at 85.6 percent, Zamfara at 83.7 percent, Plateau at 82.6 percent, Abia at 82.5 percent, Taraba at 81.9 percent, and Enugu at 81.9 percent. A national response that treats this variation as a single homogeneous crisis misallocates both food and transfer budgets.

Household-resolution prediction would meaningfully change targeting. Nigeria’s largest social-protection programmes, including the National Social Safety Nets Project and state-level cash transfer schemes, use variants of proxy-means testing anchored on household characteristics collected at enrolment [2,3]. A directly fitted food-insecurity predictor using the same household covariates plus a small set of geospatial overlays, released as open code, would give researchers and programme designers a transparent alternative to proprietary targeting scores, and would make the operational trade-offs between household and contextual information quantifiable rather than assumed. Any such application is bounded by the DHS sampling frame: the model is not validated for conflict-displaced households in camps or informal settlements (see Limitations).

Machine-learning methods are well suited to this task. Tree ensembles absorb non-linearities, pairwise and higher-order interactions, and highly-skewed predictor distributions without requiring functional-form assumptions [4,5]. They typically beat well-specified logistic regression on tabular survey data when the outcome is common and the feature set is moderate [6]. Critically, they are now accompanied by exact post-hoc interpretation methods such as SHAP [7,8] that expose the contribution of each feature to each prediction. The opacity objection that earned tree ensembles a reputation for black-box unsuitability in applied settings is much weaker in 2026 than it was in 2015.

A parallel strand of food-security research has assembled high-resolution geospatial overlays from satellite and event data, including CHIRPS rainfall [9], MODIS vegetation indices [10], ACLED conflict event records [11], WorldPop population density [12], and travel-time surfaces built on the Malaria Atlas Project friction model [13]. These products have been used to predict population-level food insecurity in Malawi [14] and in multi-country panels produced for the World Bank early-warning programme [15]. Zhou et al. [16] have articulated the principles that methodological papers in this literature should satisfy, including transparency of modelling choices, uncertainty quantification, and reproducible pipelines. These geospatial products have not yet been combined with NDHS household data in a single open, reproducible machine-learning pipeline for Nigeria.

This paper contributes such a pipeline and reports its empirical findings. Four specific contributions are claimed. First, we build and openly release an end-to-end machine-learning pipeline that predicts moderate-or-severe household food insecurity in Nigeria from the 2024 NDHS FIES module combined with five open geospatial layers, with fixed random seed, pinned dependencies, and a deposited reproducibility package. Second, the SHAP decomposition of the fitted ensembles, evaluated under cluster-grouped splits that prevent enumeration-area information from leaking across the train-test boundary, assigns between 42 and 52 percent of predictive signal to contextual predictors, a materially larger share than the 35–45 percent reported in earlier DHS tree-ensemble work [17–19]. Third, we document an association between longer travel time to the nearest city and lower food-insecurity risk, replicated across the tree ensemble and a multilevel logistic regression, presented as a subsistence-buffer hypothesis to be tested in subsequent survey rounds. Fourth, we document how a household-level random split of a clustered survey inflates ensemble performance estimates, and release the cluster-grouped evaluation design as part of the pipeline. The Methods section describes the data and the modelling pipeline. The Results section reports performance, SHAP decompositions, and partial-dependence findings. The Discussion engages the implications and limitations.

## Methods

### Ethics statement

This study is a secondary analysis of de-identified public-use microdata from the 2024 Nigeria Demographic and Health Survey (NDHS) and five open geospatial datasets. No primary data collection was undertaken. The original 2024 NDHS survey methodology and instruments received clearance from the ICF Institutional Review Board (Rockville, Maryland, USA) and the National Health Research Ethics Committee of Nigeria [1]. Informed consent from all survey respondents was obtained at the point of interview by National Population Commission field teams following the standard DHS Program protocol. The NDHS microdata are released by the DHS Program under a data-use agreement that covers academic secondary research; the geospatial covariates are open data products under licences permitting academic use. The 2024 NDHS microdata were accessed from the DHS Program on 14 January 2026. The authors did not have access to any information that could identify individual participants: the DHS Program de-identifies all microdata before release, and cluster GPS coordinates are randomly displaced 2–10 km to prevent re-identification. No additional institutional ethics review was required for this secondary analysis because the data are anonymised at source and publicly released for secondary analytical use.

### Data sources and analytical sample

This study draws on the 2024 Nigeria Demographic and Health Survey, the first round of the Nigerian DHS series to include the Food Insecurity Experience Scale [20,21]. The survey was implemented by the National Population Commission, Federal Ministry of Health and Social Welfare, and ICF and produced a national estimate of 67.7 percent of the de jure population experiencing moderate-or-severe food insecurity and 33.0 percent experiencing severe food insecurity [1]. The analytical sample comprises 39,974 households from 1,380 enumeration area (EA) clusters after dropping 73 observations with missing education of the household head.

Five open geospatial layers were joined to NDHS cluster GPS coordinates. Rainfall anomalies were computed from CHIRPS v2.0 [9] as the percent deviation of survey-year rainfall (January to December 2023) from the preceding twelve months (December 2021 to November 2022) at each cluster centroid. Vegetation anomalies were derived analogously from MODIS MOD13A2 NDVI composites at 1 km resolution exported from Google Earth Engine [10], with the survey year taken as December 2022 to November 2023 and the baseline as the preceding twelve months (December 2021 to November 2022). Conflict exposure was the natural log of one plus the count of events recorded by ACLED within a 50 km buffer of each cluster across the December 2021 to May 2024 survey window [11]. Population density was read from WorldPop 2020 constrained estimates within the smallest data-bearing window around the centroid [12]. Travel time to the nearest city of one to five million inhabitants was read from the global accessibility layers of Nelson et al. [22], which are computed on the Malaria Atlas Project friction surface [13]. DHS GPS coordinates were used as-issued without reversing the 2–10 km random displacement, because this displacement is small relative to the 50 km conflict buffer and the kilometre-scale resolution of the climate layers [23].

### Outcome variable

The outcome is a binary indicator of moderate-or-severe household food insecurity derived from the FIES. The FIES is an eight-item experiential scale developed by FAO and calibrated to a global reference metric through the Rasch model [21]. It formed part of the 2024 NDHS Household Questionnaire and was answered by the household respondent, typically the head or a designated adult member of the household. Each item asks whether, in the preceding twelve months, any member of the household experienced the specific food-insecurity experience described. Item responses were calibrated to the FAO global reference scale by FMoHSW, NPC, and ICF following the FAO protocol [20], producing a continuous probability that the household sits at or above the moderate-or-severe threshold. Households were classified as food insecure when this probability crossed the FAO cut-point, yielding the national prevalence of 67.7 percent cited above.

### Predictor set

Fifteen predictors entered the models, grouped into two blocks for the decomposition reported below. The household block contains seven variables: household size, sex of head (female indicator), age of head, education of head (ordinal: none, primary, secondary, higher), wealth quintile, count of children under five, and count of members aged 65 and above. The contextual block contains eight variables: rural-residence indicator, cluster longitude, cluster latitude, log population density, travel time to nearest city in hours, 12-month rainfall anomaly in percent, 12-month NDVI anomaly in percent, and the log count of ACLED conflict events within 50 km. Geographic coordinates were retained so that the learner could absorb residual spatial structure not carried by the five geospatial covariates. We selected this set because every variable is already available at the household level in the NDHS or is refreshable without new fieldwork once the geospatial inputs are updated, and each variable has a direct policy analogue: wealth proxies income-based targeting, conflict and travel time proxy logistical targeting, and rainfall and NDVI anomalies proxy climate-shock targeting. No lagged FIES variable is available, because 2024 is the first NDHS round to include the module.

### Model specification

Two tree-based ensembles were fit as the main predictive models: Gradient-Boosted Trees (GBT) [4] and Random Forest (RF) [5], both implemented in scikit-learn 1.3 [24]. Hyperparameters followed conventional defaults for tabular classification at this sample size, fixed at: 300 trees, maximum depth 5, learning rate 0.1, subsample 0.8 for GBT; 200 trees, maximum depth 12, and minimum five samples per leaf for RF. Hyperparameters were fixed a priori and not tuned; five-fold cross-validation on the training partition, with outcome-stratified and cluster-grouped folds (StratifiedGroupKFold), reports their stability across resamples (S1 Table). Because the NDHS draws roughly 29 households per enumeration area and all contextual predictors are constant within an enumeration area, a household-level random split would place households from nearly every cluster in both training and test sets, allowing flexible learners to memorise cluster-level outcome prevalence rather than generalise. The sample was therefore split at the enumeration-area level by grouped random assignment (GroupShuffleSplit on the DHS cluster identifier, hv001): 1,104 enumeration areas (31,975 households) form the training set and 276 enumeration areas (7,999 households) the held-out test set, with no enumeration area contributing households to both. For comparison, we also report the conventional household-level stratified split, which quantifies the performance inflation that enumeration-area leakage produces. All random seeds were fixed at a single value documented in the reproducibility package, allowing exact replication of the train-test split, the bootstrap resampling, and the SHAP computation.

A two-level logistic regression with EA-level random intercepts was fit as an inferential benchmark, estimated by adaptive Gaussian quadrature [25]. Its coefficients and intraclass correlations quantify design-respecting associations on the full sample; its purpose is variance decomposition and effect estimation, not prediction, so its held-out discrimination is not reported. Out-of-sample discrimination comparisons use a single-level logistic regression fit on the identical grouped training split as the ensembles. The multilevel model produces the intraclass correlation needed to quantify how much food-insecurity risk operates at the cluster rather than the household level.

### Model interpretation

Tree ensembles are not directly interpretable without an explanation layer. We use SHAP values [7] computed through the TreeExplainer algorithm, which produces exact Shapley decompositions for tree models in polynomial time [8]. Three diagnostics are produced. A global SHAP summary ranks features by mean absolute SHAP on the test set and shows the direction of each feature’s contribution. Partial dependence plots show the marginal effect of the top features on predicted risk. A block-level decomposition partitions total mean absolute SHAP across the household and contextual blocks, yielding the share of predictive signal attributable to each block. We report the decomposition for both GBT and RF to confirm that the finding is model-agnostic. Three illustrative local explanations are shown below for households drawn from Yobe, Lagos, and Plateau.

### Evaluation

Model performance was assessed on the held-out 20 percent test set using AUROC, sensitivity, specificity, F1, and the Brier score for calibration. Ninety-five percent confidence intervals for AUROC were produced by a 1,000-iteration cluster bootstrap that resamples enumeration areas with replacement, retaining all households of each sampled area; household-level resampling treats clustered observations as independent and understates interval width. The deployment threshold for sensitivity and specificity was set at the probability that maximised the Youden J statistic on the training set, preventing threshold selection from leaking test-set information. Calibration was assessed by binning test-set predictions into deciles of predicted risk and plotting mean predicted probability against observed prevalence within each bin. The train-test AUROC gap is reported with its pre-specified 0.15 trigger, but this diagnostic is structurally unable to detect cluster-level memorisation, because memorised cluster prevalence generalises to held-out households from the same clusters; the grouped split, not the gap diagnostic, is the safeguard against that failure mode. As a stricter test matching the targeting use case of scoring areas the survey never sampled, we report leave-zone-out validation, training on five geopolitical zones and scoring the sixth, rotated across all six zones. All models were refit on four feature sets (full, without raw coordinates, household-only, contextual-only) to separate block contributions from coordinate effects. Performance at the training-frozen Youden threshold is additionally reported by geopolitical zone, urban-rural residence, and sex of household head, with calibration-in-the-large per subgroup. Where imputation is required it uses training-fold medians only. Accumulated local effects (ALE), which estimate feature effects without extrapolating into unpopulated regions of the feature space, complement the partial-dependence plots for the top-ranked features.

A note on survey weights. The machine-learning task operates on the unweighted household sample: the estimand is household-level risk ranking within the surveyed population, and DHS sampling weights exist to correct design-based prevalence estimation, not within-sample discrimination. Design-weighted prevalence figures cited from the NDHS Final Report are flagged as such wherever they appear. The multilevel benchmark accounts for the clustering induced by the two-stage sample through its enumeration-area random intercepts; sampling weights and stratification are not incorporated in that model.

## Results

### Sample characteristics

The analytical sample of 39,974 households covered all 36 states plus the Federal Capital Territory, with 1,380 EA clusters distributed in proportion to NDHS sampling weights. Twenty-one percent of households were female-headed. Wealth quintiles were distributed as designed by DHS. Rural and urban residence were nearly balanced in the analytical sample. The outcome variable, moderate-or-severe food insecurity, was present in 68.8 percent of households at the unweighted household basis (27,508 of 39,974). This differs slightly from the 67.7 percent headline prevalence reported in the NDHS 2024 Final Report [1] because that figure is computed on a design-weighted de jure person basis, whereas the ML task operates on an unweighted household basis. Table 1 summarises the predictors and the outcome by food-insecurity status.

**Table 1:** Characteristics of the analytical sample, by food-insecurity status (n = 39,974 households from 1,380 enumeration-area clusters).

Variable	All households	Food secure	Food insecure^a^
Households, n	39,974	12,466	27,508
Household size, mean (SD)	4.39 (3.06)	4.27 (3.08)	4.45 (3.04)
Age of household head, years, mean (SD)	45.3 (15.6)	45.3 (15.8)	45.3 (15.5)
Female-headed household, %	21.1	17.4	22.7
*Education of head, n (%)*			
None	10,378 (26.0)	3,014 (24.2)	7,364 (26.8)
Primary	6,703 (16.8)	1,545 (12.4)	5,158 (18.8)
Secondary	13,910 (34.8)	3,943 (31.6)	9,967 (36.2)
Higher	8,983 (22.5)	3,964 (31.8)	5,019 (18.2)
*Wealth quintile, n (%)*			
Q1 (poorest)	6,856 (17.2)	1,733 (13.9)	5,123 (18.6)
Q2	6,242 (15.6)	1,612 (12.9)	4,630 (16.8)
Q3	7,769 (19.4)	1,876 (15.0)	5,893 (21.4)
Q4	9,187 (23.0)	2,474 (19.8)	6,713 (24.4)
Q5 (richest)	9,920 (24.8)	4,771 (38.3)	5,149 (18.7)
Children under 5, mean (SD)	0.67 (0.94)	0.63 (0.91)	0.68 (0.95)
Members aged 65 or above, mean (SD)	0.17 (0.43)	0.18 (0.44)	0.17 (0.43)
Rural residence, %	49.5	47.4	50.5
Travel time to nearest city, hours, mean (SD)	1.61 (1.52)	1.63 (1.50)	1.60 (1.53)
Log ACLED events within 50 km, mean (SD)^b^	5.25 (1.31)	5.25 (1.32)	5.25 (1.31)
Rainfall anomaly, %, mean (SD)^c^	9.46 (14.68)	9.27 (13.40)	9.55 (15.22)
NDVI anomaly, %, mean (SD)^d^	7.80 (11.04)	7.56 (10.81)	7.91 (11.14)
Log population density, mean (SD)^e^	3.43 (0.84)	3.43 (0.85)	3.43 (0.84)

^a^Moderate-or-severe food insecurity at the household level as classified by the FAO Rasch probability threshold applied to the NDHS 2024 FIES module [1].

^b^Natural logarithm of the count of ACLED events within a 50 km buffer of the cluster centroid across the December 2021 to May 2024 survey window (one added to handle zero counts).

^c^Percent deviation of survey-year (January to December 2023) cluster-centroid CHIRPS rainfall from the preceding twelve months (December 2021 to November 2022).

^d^Percent deviation of survey-year (December 2022 to November 2023) MODIS MOD13A2 NDVI from the preceding twelve months (December 2021 to November 2022) at the cluster centroid.

^e^Natural logarithm of the WorldPop 2020 constrained population density at the cluster centroid (persons per square kilometre, plus one).

### Model performance

Table 2 compares the two tree ensembles and the single-level logistic regression under the cluster-grouped split, alongside the household-level split reported for comparison. On the held-out test set of 276 enumeration areas (7,999 households), Random Forest reached AUROC 0.727, cluster-bootstrap 95 percent CI [0.704, 0.747], with a Brier score of 0.185. Gradient-Boosted Trees reached AUROC 0.714 [0.690, 0.737], Brier 0.188. The single-level logistic regression returned AUROC 0.679 [0.653, 0.702], Brier 0.195 under the same grouped split. The gradient-boosted and logistic confidence intervals overlap; the random-forest and logistic intervals are marginally disjoint. The ensembles retain an advantage of 3.5 to 4.8 AUROC points within surveyed clusters. For comparison, a conventional household-level random split of the same data yields AUROC 0.773 for GBT against 0.681 for the logistic baseline, a 9.2-point margin; the contrast between the two designs quantifies the enumeration-area information leakage that household-level splits permit (see Methods). Grouped five-fold cross-validation on the training partition gave mean AUROC 0.729 (SD 0.009) for GBT, 0.731 (SD 0.008) for RF, and 0.681 (SD 0.012) for the logistic regression, confirming that the fixed hyperparameters are stable across resamples. The multilevel logistic regression produced an intraclass correlation of 0.235 in the null specification and 0.206 in the full-covariate specification, indicating that roughly one-fifth of residual variation in food-insecurity risk operates at the cluster level after household and contextual covariates are accounted for. Under the grouped split the train-test AUROC gap was 0.160 for GBT, above the pre-specified 0.15 trigger, and 0.133 for RF; as described in Methods, this diagnostic cannot detect the leakage that the grouped design removes, and we report it for completeness rather than as the operative safeguard. Consistent with the pre-specified rule, the conclusions of this paper rest on the random forest and on ranges across models rather than on the gradient-boosted model alone. Feature-set ablations under the grouped split give AUROC 0.692 (GBT) and 0.700 (RF) without raw coordinates, 0.625 and 0.638 for household-only models, and 0.635 and 0.647 for contextual-only models, with the logistic baseline at 0.662, 0.625, and 0.568 respectively; household-only and contextual-only blocks are close to interchangeable in discriminative power. Table 2 summarises test-set performance across models.

**Table 2:** Test-set performance across models (n = 7,999 held-out households in 276 enumeration areas; cluster-grouped split).

Metric	GBT	RF	Single-level LR	Multilevel LR^a^
AUROC, test	0.714	0.727	0.679	—
AUROC, 95 percent CI^b^	[0.690, 0.737]	[0.704, 0.747]	[0.653, 0.702]	—
Brier score, test^c^	0.188	0.185	0.195	—
Sensitivity at Youden threshold^d^	0.712	0.670	0.583	—
Specificity at Youden threshold	0.594	0.651	0.669	—
F1 at Youden threshold	0.751	0.733	0.673	—
Youden-J threshold (training set)	0.672	0.689	0.700	—
ICC, null specification	—	—	—	0.235
ICC, full-covariate specification	—	—	—	0.206
Train–test AUROC gap^e^	0.160	0.133	—	—

^a^Two-level logistic regression with enumeration-area random intercepts (Stata adaptive Gaussian quadrature). ICC only; AUROC not directly comparable because the model was fit on the full sample.

^b^1,000-iteration cluster bootstrap of the held-out test set, resampling enumeration areas with replacement and retaining all households of each sampled area.

^c^At the 0.688 base rate, no-skill Brier = 0.215; Brier skill scores: RF 14 percent, GBT 12 percent, LR 9 percent.

^d^Probability cut-off maximising Youden J on training set; fixed before test-set evaluation.

^e^Train AUROC minus test AUROC; > 0.15 pre-specified as a re-specification trigger. The grouped-split GBT gap exceeds the trigger; as described in Methods, this diagnostic cannot detect cluster-level memorisation and the grouped split is the operative safeguard.

At the Youden-J optimal threshold (0.672 for GBT, 0.689 for RF, both fit on the training set), GBT produced test-set sensitivity 0.712 and specificity 0.594, and RF sensitivity 0.670 and specificity 0.651 (Table 2). The Youden threshold trades off the two error rates equally, and for an outcome with 69 percent prevalence the optimal cut-off lies well above the 0.5 default. Subgroup results, reported below, show that a single pooled threshold produces uneven error profiles across zones, so operators should expect to set zone-specific thresholds.

### SHAP feature importance

Fig 1 presents the SHAP summary for the GBT ensemble under the cluster-grouped evaluation. Wealth quintile was the dominant single predictor, contributing 30.6 percent of total mean absolute SHAP across the test set. The next four features were all contextual: latitude (12.1 percent), travel time to the nearest city (8.7 percent), longitude (7.7 percent), and rainfall anomaly (7.3 percent). Household size, log conflict events within 50 km, log population density, age of household head, and the rural-residence indicator completed the top ten, each contributing between 3.5 and 6.8 percent. The remaining five variables (NDVI anomaly, education of household head, female-headship, count of children under five, count of elderly members) each contributed 2.9 percent or less. Latitude and longitude jointly carry 19.8 percent, essentially unchanged from the household-split model, which illustrates that SHAP shares alone do not diagnose evaluation-design leakage. Table 3 reports the full feature ranking and Fig 1 shows the corresponding beeswarm.

**Table 3:** SHAP feature importance ranking for the Gradient-Boosted trees ensemble, computed on the cluster-grouped held-out test set (n = 7,999).

Rank	Feature	Mean |SHAP| contribution, %^a^	Block
1	Wealth index	30.6	Household
2	Cluster latitude	12.1	Contextual
3	Travel time to nearest city	8.7	Contextual
4	Cluster longitude	7.7	Contextual
5	Rainfall anomaly, 12 months	7.3	Contextual
6	Household size	6.8	Household
7	Log ACLED events within 50 km	5.5	Contextual
8	Log population density	4.6	Contextual
9	Age of household head	3.9	Household
10	Rural residence	3.5	Contextual
11	NDVI anomaly, 12 months	2.9	Contextual
12	Education of household head	2.8	Household
13	Female-headed household	2.4	Household
14	Children under 5	0.8	Household
15	Members aged 65 or above	0.4	Household

^a^Percent of total mean absolute SHAP across the test set; values sum to 100 percent.

**Fig 1:**
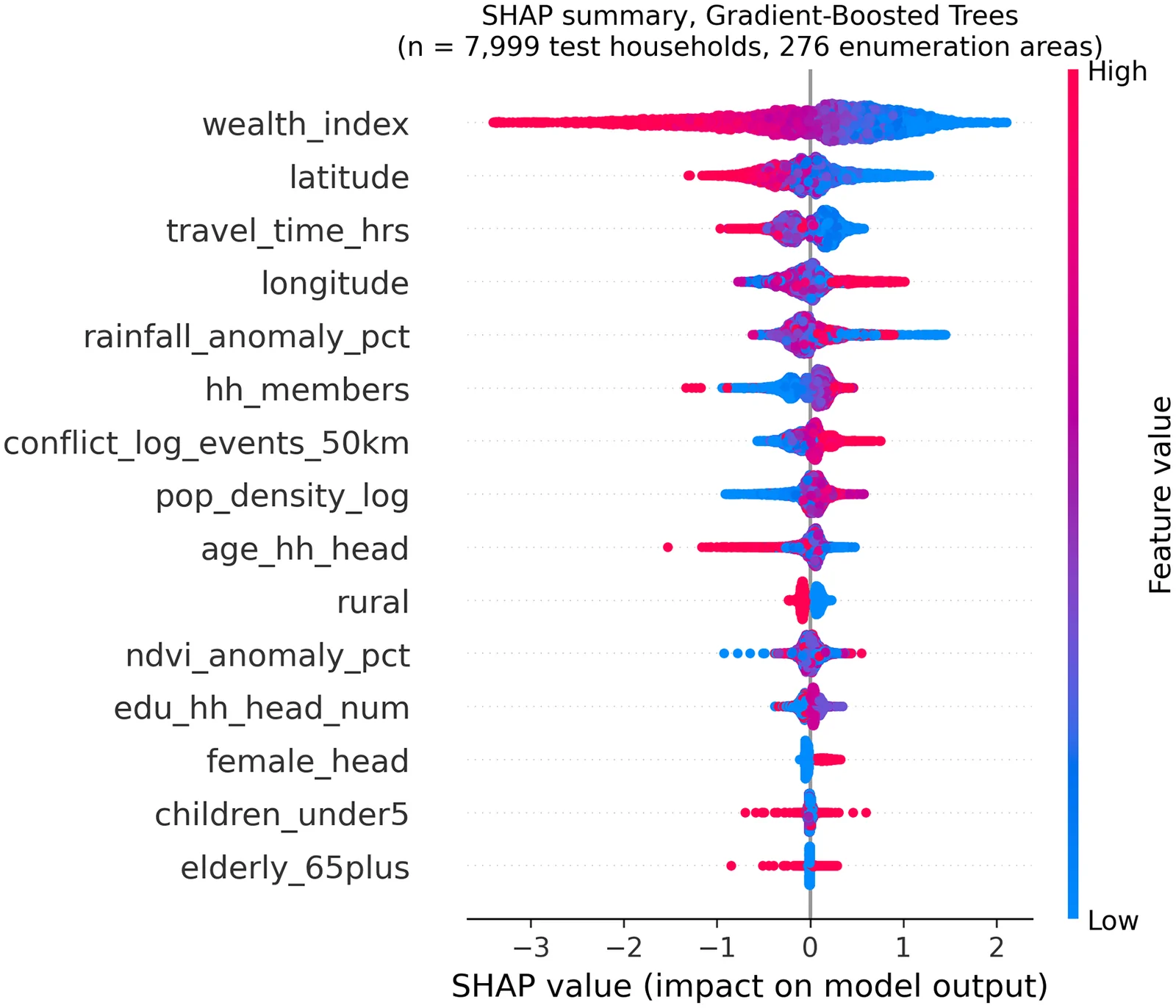
SHAP summary plot for the gradient-boosted trees ensemble on the cluster-grouped held-out test set (n = 7,999 households from 276 enumeration areas). Features are ordered top to bottom by mean absolute SHAP contribution. Each point represents one household; the horizontal axis shows the SHAP contribution to the predicted probability of moderate-or-severe food insecurity; colour shows the feature value (red high, blue low).

The direction of each feature’s SHAP contribution (visible in the beeswarm) matched economic expectation for most variables. Higher wealth quintile reduced predicted risk. Longer travel time to the nearest city reduced predicted risk. Higher rainfall deviation from the long-run mean increased predicted risk, consistent with both drought and flood shocks. Higher conflict counts within 50 km increased predicted risk. Rural residence increased predicted risk relative to urban. Higher education of the household head reduced predicted risk, though the effect was smaller than that of wealth. The NDVI anomaly direction was weak and close to zero, matching the non-significance of the NDVI coefficient in the multilevel regression (OR 1.023, 95 percent CI 0.965 to 1.084, p = 0.448).

### Household versus contextual decomposition

Table 4 reports the block-level SHAP decomposition under the cluster-grouped evaluation, with cluster-bootstrap 95 percent confidence intervals on the household share. In the GBT ensemble the household block accounted for 47.7 percent of total mean absolute SHAP (CI 46.4 to 49.1) and the contextual block for 52.3 percent; in the RF ensemble the split was 51.3 (CI 49.7 to 53.2) against 48.7 percent. Removing raw coordinates shifts the decomposition toward the household block: 51.9 percent (CI 50.6 to 53.2) in GBT and 57.6 percent (CI 56.3 to 59.0) in RF. Joint block-permutation importance, a SHAP-free check, weights the household block more heavily still: permuting the household block drops grouped-split GBT AUROC by 0.168 against 0.121 for the contextual block. Across specifications the contextual share spans 42–52 percent, against the 35–45 percent typical of earlier SHAP-based food-security work using Demographic and Health Surveys [17,18]; the two attribution methods bound the answer from opposite directions. Wealth quintile alone still accounts for 31 percent of total signal, so household characteristics are not less important than previously thought. The combined predictive weight of the contextual variables, once exposure to conflict, climate anomaly, travel time, rural residence, and population density are taken together, approaches the weight of the seven household variables. Geographic targeting and household targeting, in other words, remain complementary rather than substitutable for Nigeria.

**Table 4:** Predictive-signal decomposition across the household and contextual predictor blocks, both ensembles. Cluster-grouped evaluation; full feature set and without raw coordinates.

Predictor block^a^	GBT, % of total |SHAP|	RF, % of total |SHAP|
Household (7 variables), full set^b^	47.7	51.3
Contextual (8 variables), full set^c^	52.3	48.7
Household, without coordinates	51.9	57.6
Contextual, without coordinates	48.1	42.4

^a^Shares are the sum of mean absolute SHAP contributions of the variables assigned to each block, divided by the model-wide total and multiplied by 100.

^b^Household block: wealth index, household size, female-head indicator, age of head, education of head, count of children under 5, count of members aged 65 or above.

^c^Contextual block: rural-residence indicator, cluster latitude, cluster longitude, log population density, travel time to nearest city, log ACLED events within 50 km, 12-month rainfall anomaly, 12-month NDVI anomaly.

^d^Cluster-bootstrap 95 percent CIs for the household share: full set, GBT 46.4 to 49.1, RF 49.7 to 53.2; without coordinates, GBT 50.6 to 53.2, RF 56.3 to 59.0. Joint block-permutation AUROC drops, full-set GBT: household 0.168, contextual 0.121.

### Spatial generalization

Table 5 reports leave-zone-out validation, the design that matches the targeting use case of scoring areas the survey never sampled. The logistic baseline outperformed the gradient-boosted ensemble in all six geopolitical zones, with mean AUROC 0.671 against 0.628. Zone-level discrimination ranged from 0.576 (GBT, North West held out) to 0.750 (logistic regression, South South held out). For extrapolation to unsampled areas, the ensemble advantage observed within surveyed clusters does not merely shrink; it reverses. The ensembles’ within-survey advantage evidently reflects local structure that does not transfer across zones, and the simpler model travels better. Any deployment that scores unsurveyed areas should weigh this result heavily.

**Table 5:** Leave-zone-out spatial validation. Each row trains on five geopolitical zones and scores the held-out sixth (full feature set). The logistic baseline outperforms the gradient-boosted ensemble in every zone.

Held-out zone	n	Prevalence	GBT AUROC	LR AUROC
North Central	7,615	0.650	0.616	0.619
North East	5,964	0.764	0.588	0.622
North West	7,586	0.630	0.576	0.658
South East	5,291	0.733	0.653	0.714
South South	6,377	0.705	0.696	0.750
South West	7,141	0.679	0.635	0.662
Mean	—	—	0.628	0.671

### Subgroup performance

Table 6 reports GBT performance at the training-frozen Youden threshold (0.672) by geopolitical zone, urban-rural residence, and sex of household head on the grouped holdout. Discrimination ranges from AUROC 0.674 in the North Central zone to 0.756 in the South East. Sensitivity at the pooled threshold falls to 0.622 in the North West, the subgroup where false negatives concentrate, and specificity falls to 0.413 in the North East, where prevalence is highest and false positives concentrate. Calibration-in-the-large stays within plus or minus 0.031 across all ten subgroups. Because the outcome’s prevalence spans 37.9 to 94.1 percent across states, pooled metrics conceal exactly where classification errors concentrate; the subgroup table makes those asymmetries explicit and should inform any threshold choice in application. The model is not validated for conflict-displaced households in camps or informal settlements, which the DHS sampling frame excludes.

**Table 6:** Subgroup performance of the GBT ensemble at the training-frozen Youden threshold (0.672) on the cluster-grouped holdout. CITL is calibration-in-the-large, observed prevalence minus mean predicted probability.

Subgroup	n	Prevalence	AUROC	Sensitivity	Specificity	CITL
North Central	1,395	0.672	0.674	0.631	0.596	+0.016
North East	1,272	0.751	0.738	0.848	0.413	−0.031
North West	1,546	0.622	0.690	0.622	0.671	−0.018
South East	903	0.734	0.756	0.833	0.525	−0.004
South South	1,278	0.691	0.723	0.701	0.633	−0.004
South West	1,605	0.682	0.697	0.679	0.618	+0.017
Urban	4,407	0.673	0.726	0.694	0.639	+0.001
Rural	3,592	0.704	0.697	0.734	0.533	−0.009
Male-headed	6,277	0.671	0.710	0.691	0.608	−0.008
Female-headed	1,722	0.746	0.715	0.781	0.526	+0.014

### Partial dependence and the travel-time paradox

Fig 2 shows partial dependence plots (PDP) for the four highest-SHAP features. The wealth PDP is monotonically protective, consistent with entitlement theory [26]: moving across the observed range of the wealth index reduces predicted probability of moderate-or-severe food insecurity by roughly 0.61 in the GBT ensemble, averaged over the observed distribution of the remaining features.

**Fig 2:**
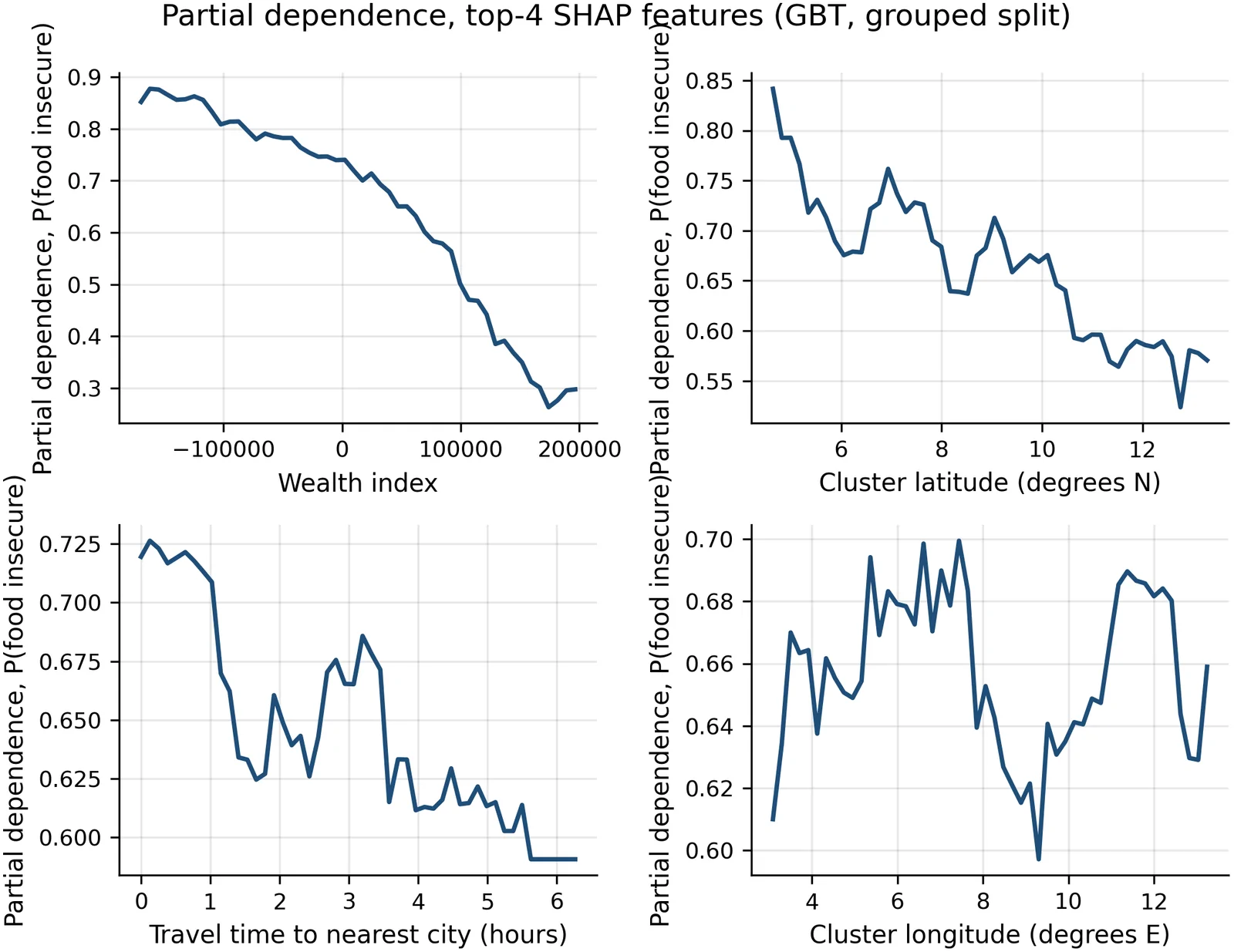
Partial dependence plots for the four highest-SHAP features in the gradient-boosted trees ensemble: wealth index, cluster latitude, travel time to nearest city, and cluster longitude. Each panel shows the marginal effect of the feature on the predicted probability of moderate-or-severe food insecurity, averaged over the observed distribution of the remaining features. Horizontal axes are in original feature units.

The travel-time PDP shows the pattern that was already visible as an inverted coefficient in the multilevel regression (OR 0.790, 95 percent CI 0.734 to 0.849, p < 0.001): longer travel time to the nearest city of one to five million inhabitants is associated with lower predicted probability of food insecurity, not higher. This result runs against the assumption embedded in many market-access theoretical frames, which treats physical remoteness from urban markets as a driver of food insecurity [27]. The direction holds in our data. Because partial dependence can extrapolate into unpopulated regions of a correlated feature space, we verified the pattern with accumulated local effects (S1 Fig): the ALE curve is positive for households within roughly one hour of a city, crosses zero near 1.1 hours and is negative thereafter, apart from a brief positive excursion around three hours, so the association is concentrated in the contrast between near-city and remote households rather than manufactured by extrapolation. We advance a subsistence-buffer hypothesis: in the Nigerian context, more remote rural households retain larger shares of own-production in their food basket and are less exposed to cash-market price shocks, which in periods of sharp currency depreciation and fuel-price inflation would become a risk-reducing channel [28]. The near-city elevation of risk in the ALE curve is consistent with this market-exposure reading, but a single cross-section cannot establish the mechanism. We return to this in the Discussion. Whether the association varies across wealth and residence subgroups is not tested here; wealth-stratified and residence-stratified estimation is a priority for future validation.

### Calibration

Fig 3 shows the calibration curve for the GBT ensemble on the held-out test set. The curve tracks the 45-degree line across most of the predicted-probability range, with modest over-prediction in the 0.3 to 0.5 band (predicted probabilities in this band slightly exceed the observed prevalence in the corresponding deciles) and good agreement in the 0.6 to 0.9 band where most households sit. The Brier score on the grouped test set was 0.185 for RF and 0.188 for GBT, against 0.195 for the single-level logistic baseline and 0.215 for a no-skill predictor at the unweighted household base rate of 0.688. Expressed as a Brier skill score relative to the no-skill reference, RF delivers a 14 percent reduction in mean squared probabilistic error (GBT 12 percent; LR 9 percent); the probabilistic gains over the linear baseline are real but modest under leakage-free evaluation. Calibration-in-the-large is within 0.003 of zero for all three models. Calibration is an operational requirement for deployment, because risk scores that are miscalibrated cannot be compared across districts or programmes. The curve suggests that post-hoc calibration via isotonic regression is not needed for the GBT ensemble in this application, though re-calibration before production deployment is a standard safeguard [29].

**Fig 3:**
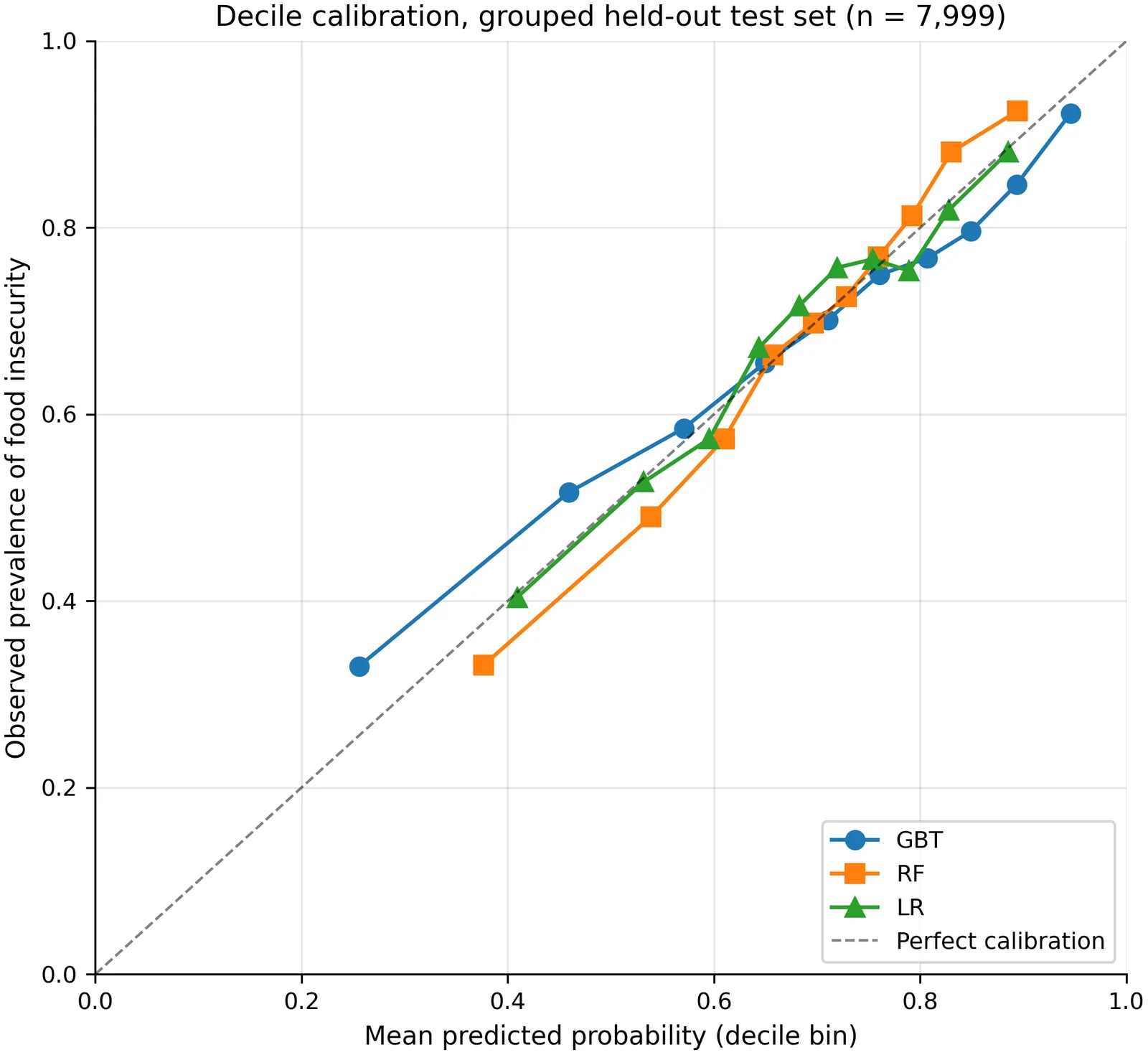
Decile calibration curve on the cluster-grouped held-out test set (n = 7,999). The horizontal axis is the mean predicted probability within each decile bin; the vertical axis is the observed prevalence of moderate-or-severe food insecurity within the same bin. Markers correspond to the three classifiers (GBT, RF, single-level logistic regression). The dashed line indicates perfect calibration.

### Illustrative local explanations

Three test-set households illustrate how the model explains individual predictions (Fig 4). A high-risk household in Yobe with a large positive rainfall anomaly, low wealth, and a far north-eastern location reaches a predicted probability of 0.98, matching its observed food-insecure status. A low-risk household in Lagos with high wealth and an older, smaller household produces a predicted probability of 0.03, again matching the observed food-secure outcome. A mid-risk household in Plateau with mid-range wealth and low local population density produces a prediction of 0.69, near the population base rate. These per-household decompositions show how the ensemble’s reasoning can be audited one household at a time, which matters in any setting where targeting decisions need to be explainable.

**Fig 4:**
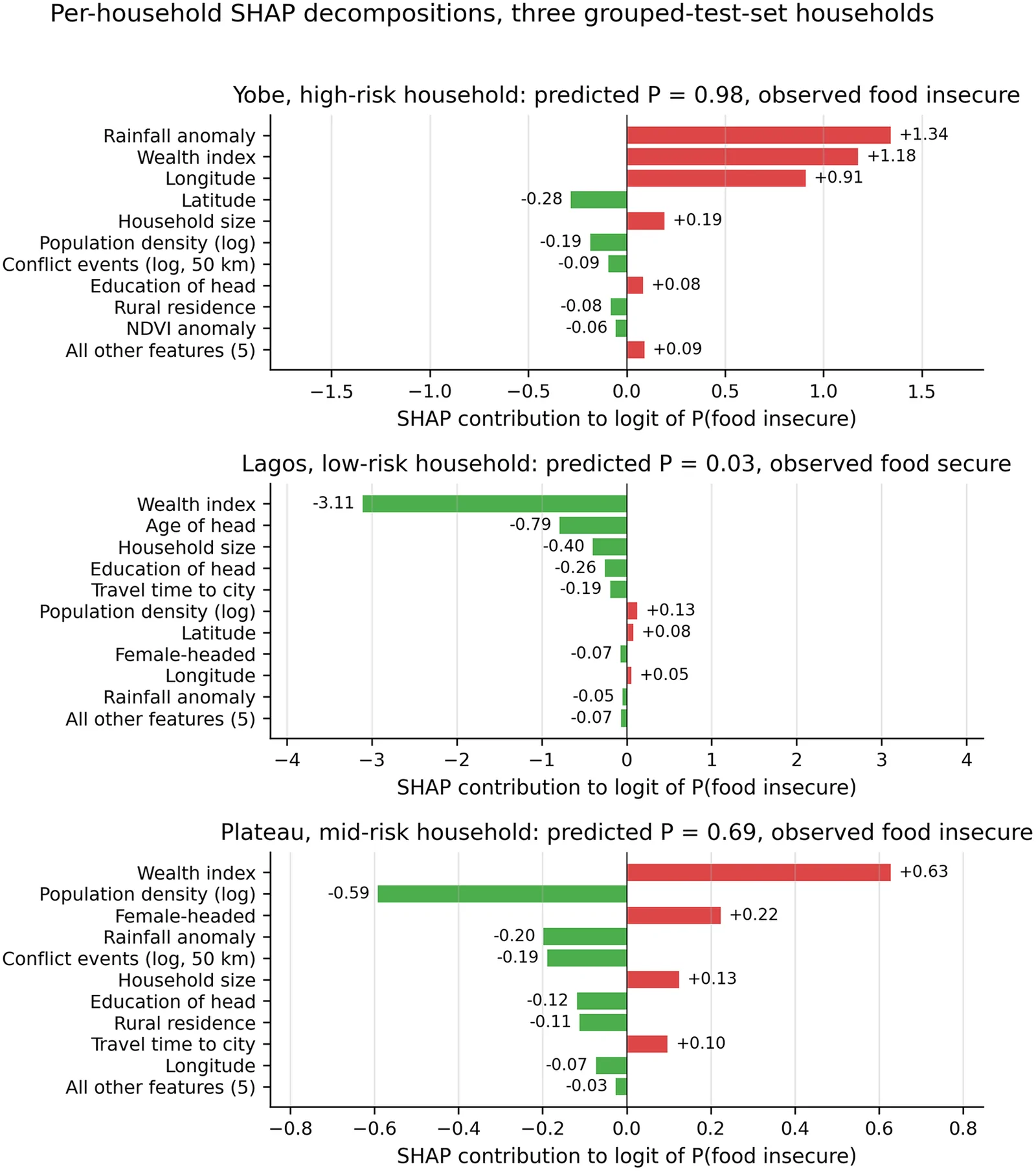
Per-household SHAP decompositions for three illustrative households from the cluster-grouped test set: a high-risk household in Yobe state (predicted probability 0.98, observed food insecure), a low-risk household in Lagos state (predicted probability 0.03, observed food secure), and a mid-risk household in Plateau state (predicted probability 0.69, observed food insecure). Bars show the SHAP contribution of each feature to the prediction on the logit scale; positive contributions increase predicted risk and negative contributions decrease it. The ten largest contributions are shown per household, with the remaining five features pooled.

## Discussion

### Reassessing the household-block dominance

The most consequential attribution result is the household-contextual balance under leakage-free evaluation. Earlier work using Demographic and Health Survey data and machine-learning feature attribution for food-security outcomes reported that household characteristics carried roughly 55–65 percent of total predictive signal in comparable tree ensembles, with the balance assigned to contextual covariates [15,17,18]. Our grouped-evaluation result for Nigeria 2024 narrows that dominance without reversing it: the household share is 47.7 percent in GBT and 51.3 percent in RF with the full feature set, rising to 51.9 and 57.6 percent when raw coordinates are removed, and the SHAP-free block-permutation check weights the household block more heavily than SHAP does. Stated as a range, contextual predictors carry 42–52 percent of predictive signal across specifications, materially above the 35–45 percent typical of the earlier literature. Part of the household dominance reported in that literature may itself reflect evaluation designs that did not group clusters, a channel our corrected design controls. The wealth quintile remains the single most important variable, which is consistent with the earlier literature, but the combined predictive weight of eight contextual variables rivals the combined weight of seven household variables.

### Evaluation design in clustered surveys

The comparison between the household-level and cluster-grouped splits carries a methodological lesson that extends beyond this paper. The NDHS, like all DHS surveys, interviews roughly 29 households per enumeration area, and every contextual covariate, including raw coordinates, is constant within an enumeration area. A household-level random split therefore places members of nearly every cluster on both sides of the train-test boundary, and a flexible learner can identify clusters through their covariate fingerprint and reproduce their training-set prevalence on the matched test households. On these data that mechanism inflated tree-ensemble AUROC by 4–6 points while moving the linear baseline by 0.2 points, manufactured a 9.2-point ensemble margin that leakage-free evaluation reduces to at most 4.8 points, and passed undetected through a train-test-gap diagnostic of the kind often reported as an overfitting safeguard. Cluster-grouped splits, grouped cross-validation, and cluster bootstraps should be standard practice for predictive work on DHS-type cluster samples. Feature-list changes such as dropping raw coordinates do not by themselves remove the leak, because the remaining cluster-constant covariates still identify clusters jointly.

### The travel-time paradox in the Nigerian context

The travel-time finding deserves separate treatment. Almost all economic models of market integration treat remoteness from urban centres as a mechanism through which rural households face higher food-price volatility and lower income opportunities [27,30]. Cadre Harmonisé analyses routinely cite poor market access as a driver of food insecurity. The empirical finding in Nigeria 2024, replicated across the tree ensemble (SHAP direction toward lower risk, third-ranked feature at 8.7 percent under grouped evaluation) and the multilevel logistic regression (OR 0.790, 95 percent CI 0.734 to 0.849, p < 0.001), points the other way. More remote households are less likely to be food insecure than their less remote peers, conditional on wealth, education, conflict exposure, and climate anomaly.

### Conflict and climate signals

Conflict exposure (log of ACLED events within 50 km) carried 5.5 percent of GBT SHAP with a strong positive direction. Rainfall anomaly carried 7.3 percent and a positive direction, consistent with both drought and flood effects increasing risk in a country that experiences both. NDVI anomaly carried only 2.9 percent and a near-zero direction. The multilevel regression confirms the weak NDVI effect (OR 1.023, 95 percent CI 0.965 to 1.084, p = 0.448). The likely explanation is that CHIRPS rainfall anomalies are upstream of NDVI in the productivity chain, so once rainfall is in the model NDVI’s residual signal is small. The 12-month NDVI aggregate may also be too coarse to capture shock timing.

### Implications for applied food-insecurity prediction

Four implications flow from these results.

First, reproducible household-level food-insecurity prediction is achievable at low cost. The deposited pipeline runs end-to-end on a laptop in under ten minutes. The inputs are the NDHS 2024 microdata and five open geospatial layers; the code is open-licensed; the random seed is fixed. This lowers the cost of replication and audit, which Zhou et al. [16] identify as pre-conditions for transparent machine-learning practice in food-security research.

Second, the household-contextual SHAP split, with contextual predictors carrying 42–52 percent of signal across specifications, implies that household-level and contextual-level predictors are complementary rather than substitutable in the Nigerian setting. Applied targeting instruments that rely only on household characteristics (for example, proxy-means tests anchored on assets and education) will miss the contextual dimension that contributes roughly half of the model’s predictive signal. Instruments that rely only on geographic aggregates will miss household heterogeneity within any given geographic unit. Where both channels are available to an analyst, both should be used.

Third, the association between longer travel time to the nearest city and lower food-insecurity risk has implications for how remoteness is treated in applied work. Longer travel times are typically entered as adverse variables in food-security scoring. Our data argue for the opposite sign in Nigeria 2024, conditional on wealth, education, conflict exposure, and climate anomaly. The finding is sensitive to the measurement choices in the DHS GPS displacement and the accessibility-layer city-size band (one to five million inhabitants), as noted in the travel-time paradox subsection above, and should be replicated in subsequent NDHS rounds before it is treated as a stable structural feature. For the present sample, the direction is consistent across the tree ensemble and the multilevel regression, and it qualifies as a finding the applied literature should not ignore.

Fourth, model choice depends on deployment geography. Within surveyed clusters the tree ensembles rank households more accurately; for extrapolation to areas the survey never sampled, the logistic model was more reliable in every zone (Table 5). National programmes covering both settings should not assume that one model serves both, and should validate whichever model they adopt under the spatial-blocking design that matches the intended use.

### Limitations

Four limitations bound the interpretation. First, the study is cross-sectional, and the results describe predictive associations at a single point in time rather than supporting causal inference. NDHS rounds are repeated cross-sections rather than household panels, since each round draws a fresh sample, so household-level longitudinal analysis is not feasible from NDHS data alone. Aggregate-level temporal analysis (at the cluster or local-government-area scale) would become possible once a second NDHS round carrying the FIES module is available. Second, the FIES outcome is an experiential measure that captures lived experience of insufficient food access over the preceding twelve months. It does not measure dietary diversity, nutrient intake, or anthropometric status, and predictions built for FIES will not transfer directly to IPC-phase or nutritional outcomes without recalibration. Third, the geospatial covariates carry measurement error from the DHS 2–10 km GPS displacement and from the accessibility-layer city-size band (one to five million inhabitants), which may attenuate or otherwise distort estimated coefficients on the spatial variables. The analysis also does not cover conflict-displaced households living in camps or informal settlements outside the DHS sampling frame, a standard limitation of DHS-based analysis in conflict-affected regions. This model should not be applied to conflict-displaced populations in camps or informal settlements without separate validation; no performance evidence exists for that population. Fourth, the machine-learning analysis is unweighted, estimating within-sample risk ranking rather than design-weighted population quantities, and exact numerical reproduction of the tree-ensemble results is architecture-specific: results reproduce exactly on identical hardware under the pinned environment, while differences in the third decimal of AUROC can appear across CPU architectures.

### Future work

Three extensions are worth pursuing. First, the construct relationship between household-level FIES predictions and area-level expert-consensus food-security classifications such as the Cadre Harmonisé and the IPC deserves systematic investigation, since the two measures address related but distinct dimensions of food insecurity at different aggregation levels. Second, the pipeline can be applied to the 2021 Nigeria MICS FIES data as a temporal-validation test, asking whether the predictor weights estimated on 2024 NDHS data hold for predicting MICS outcomes from a pre-shock period and helping triangulate which predictor effects are stable across data programmes and macroeconomic regimes. Third, when a subsequent NDHS round including the FIES module becomes available, the same out-of-sample temporal-validation procedure can be repeated on the new cross-section.

## Conclusions

This paper builds and openly releases a reproducible machine-learning pipeline for household-level food-insecurity prediction, trained on the 2024 NDHS Food Insecurity Experience Scale outcome and fifteen household and geospatial predictors, and evaluated under cluster-grouped splits that keep enumeration areas disjoint between training and test sets. Random Forest reaches AUROC 0.727 on the grouped held-out set (cluster-bootstrap 95 percent CI 0.704 to 0.747), with Gradient-Boosted Trees at 0.714 (0.690 to 0.737) and the single-level logistic regression at 0.679 (0.653 to 0.702), alongside a multilevel logistic intraclass correlation of 0.206. Wealth index is the dominant single predictor at 31 percent of total SHAP, and contextual predictors carry 42–52 percent of predictive signal across models and coordinate treatments, a larger share than comparable DHS tree-ensemble work reports. Travel time to the nearest city is associated with lower rather than higher food-insecurity risk in this sample, a pattern consistent with a subsistence-buffer channel in a year of naira depreciation and fuel-price inflation that awaits replication in the next survey round. Under leave-zone-out validation the logistic baseline outperforms both ensembles in every zone, so the ensemble advantage is a within-survey property that does not extend to unsampled areas. The methodological contributions are a transparent, auditable end-to-end pipeline in the sense of Zhou et al. [16] and a quantified demonstration that household-level splits of clustered surveys inflate ensemble performance; the empirical contributions are the contextual-share range and the travel-time association for Nigeria 2024. Code and fitted outputs are deposited at a permanent repository (DOI in the Data Availability Statement) to support replication and adaptation to other DHS-FIES countries.

## Supporting information

S1 Fig Accumulated local effects for the four highest-SHAP features. First-order ALE for wealth index, cluster latitude, travel time to nearest city, and cluster longitude, computed from the gradient-boosted trees ensemble on the cluster-grouped test set (n = 7,999 households, 276 enumeration areas) with 20 quantile bins. ALE estimates feature effects using only households observed within each bin of the feature, avoiding the extrapolation that partial dependence permits when predictors are correlated.
(TIF)

S1 Table Grouped five-fold cross-validation results. Per-fold AUROC for GBT, RF, and logistic regression under outcome-stratified, cluster-grouped folds on the training partition.
(CSV)

S1 Checklist TRIPOD-AI reporting checklist. (DOCX)
