# Supplementary material for: Household-level food-insecurity prediction from experience-scale surveys and open geospatial data: A reproducible machine-learning pipeline for Nigeria
Source: PLoS One. 2026 Oct 7;21(10):e0349978. doi: 10.1371/journal.pone.0349978 (PMC13645075; doi:10.1371/journal.pone.0349978)
Supplement: Supplementary file 1 [file pone.0349978.s003.docx]

**TRIPOD+AI Reporting Checklist**

*Household-level food-insecurity prediction from experience-scale surveys and open geospatial data: a reproducible machine-learning pipeline for Nigeria*

Philip Osung. Submitted 7 May 2026; revised 5 July 2026 (references updated to the revised manuscript)

| Section / Item | # | D/E | Where reported in manuscript |
| --- | --- | --- | --- |
| TITLE *Identify the study as developing or evaluating the performance of a multivariable prediction model, the target population, and the outcome to be predicted* | **1** | D;E | Title page (page 1). Title states 'Household-level food-insecurity prediction from experience-scale surveys and open geospatial data: a reproducible machine-learning pipeline for Nigeria.' Identifies development of a prediction pipeline; target population is Nigerian households; outcome is moderate-or-severe food insecurity. |
| ABSTRACT *See TRIPOD+AI for Abstracts checklist* | **2** | D;E | Abstract section. Reports objective, data, cluster-grouped evaluation design, key result (grouped held-out AUROC: RF 0.727, GBT 0.714, LR 0.679), and the household-versus-contextual decomposition stated as a range. |
| INTRODUCTION *Explain the healthcare context (including whether diagnostic or prognostic) and rationale for developing or evaluating the prediction model, including references to existing models* | **3a** | D;E | Introduction. Establishes context (Nigerian food insecurity, NDHS, 67.7 percent prevalence) and the prediction-model rationale (open targeting score for social-protection programmes). Cites prior ML-and-food-security work: Lentz et al. 2019 [14], Andrée et al. 2020 [15], Zhou et al. 2022 [16]. |
| *Describe the target population and the intended purpose of the prediction model in the context of the care pathway, including its intended users (e.g., healthcare professionals, patients, public)* | **3b** | D;E | Introduction. Target population: Nigerian households surveyed in the 2024 NDHS. Intended users: researchers and programme designers working on social-protection targeting (NSSNP, state-level cash-transfer schemes). Pipeline is open-source for transparent alternative to proprietary targeting scores. |
| *Describe any known health inequalities between sociodemographic groups* | **3c** | D;E | Introduction. Reports state-level prevalence inequalities (Nasarawa 37.9 percent vs Yobe 94.1 percent), highlighting spatially concentrated burden in North East, North West, and Middle Belt zones. |
| OBJECTIVES *Specify the study objectives, including whether the study describes the development or validation of a prediction model (or both)* | **4** | D;E | Introduction (last paragraph). Objective: develop and openly release a household-level food-insecurity prediction pipeline trained on the 2024 NDHS FIES module and five geospatial layers; evaluate against a held-out test set. Single-dataset internal development and evaluation. |
| METHODS — DATA *Describe the sources of data separately for the development and evaluation datasets (e.g., randomised trial, cohort, routine care or registry data), the rationale for using these data, and representativeness of the data* | **5a** | D;E | Methods. Data sources and analytical sample. Source: 2024 Nigeria Demographic and Health Survey (cross-sectional household survey, nationally representative); five open geospatial layers (CHIRPS, MODIS MOD13A2, ACLED, WorldPop, Nelson et al. 2019 travel time). Single dataset; 80/20 cluster-grouped train-test split (no enumeration area in both partitions) for development and evaluation. |
| *Specify the dates of the collected participant data, including start and end of participant accrual; and, if applicable, end of follow-up* | **5b** | D;E | Methods. Ethics statement and Data sources. NDHS 2024 fieldwork was conducted in 2024 (specific dates documented in NDHS 2024 Final Report). Microdata accessed from the DHS Program on 14 January 2026. No follow-up — cross-sectional design. |
| PARTICIPANTS *Specify key elements of the study setting (e.g., primary care, secondary care, general population) including the number and location of centres* | **6a** | D;E | Methods. Data sources and analytical sample. Setting: general-population household survey across Nigeria. 1,380 enumeration-area (EA) clusters in 36 states and the Federal Capital Territory. |
| *Describe the eligibility criteria for study participants* | **6b** | D;E | Methods. Data sources. NDHS sample frame was the Nigerian de jure household population. Analytical sample: 39,974 households after dropping 73 observations with missing education of head. |
| *Give details of any treatments received, and how they were handled during model development or evaluation, if relevant* | **6c** | D;E | N/A. Observational secondary analysis. No interventions or treatments. |
| DATA PREPARATION *Describe any data pre-processing and quality checking, including whether this was similar across relevant sociodemographic groups* | **7** | D;E | Methods. Outcome variable (FAO Rasch model for FIES); Predictor set (variable definitions, log transformations); Data sources (50 km buffer for ACLED, 12-month anomalies for CHIRPS and MODIS, GPS displacement handling). Pre-processing applied uniformly across the analytical sample. |
| OUTCOME *Clearly define the outcome that is being predicted and the time horizon, including how and when assessed, the rationale for choosing this outcome, and whether the method of outcome assessment is consistent across sociodemographic groups* | **8a** | D;E | Methods. Outcome variable. Binary indicator of moderate-or-severe household food insecurity, derived from the eight-item Food Insecurity Experience Scale (FIES) using the FAO Rasch probability threshold. Assessed at the time of NDHS interview. Consistent definition across all households. |
| *If outcome assessment requires subjective interpretation, describe the qualifications and demographic characteristics of the outcome assessors* | **8b** | D;E | N/A. FIES is a self-report instrument; no third-party assessor. Items were administered by trained NDHS interviewers following the standardised DHS Program protocol. |
| *Report any actions to blind assessment of the outcome to be predicted* | **8c** | D;E | N/A. Self-reported outcome; blinding not applicable in observational survey design. |
| PREDICTORS *Describe the choice of initial predictors (e.g., literature, previous models, all available predictors) and any pre-selection of predictors before model building* | **9a** | D | Methods. Predictor set. Fifteen predictors selected on theoretical and prior-literature grounds: seven household features (wealth, household size, sex/age/education of head, children under 5, members 65+) and eight contextual features (rural residence, latitude, longitude, log population density, travel time, log ACLED events, rainfall and NDVI anomalies). No pre-selection by univariate screening. |
| *Clearly define all predictors, including how and when they were measured (and any actions to blind assessment of predictors for the outcome and other predictors)* | **9b** | D;E | Methods. Predictor set and Data sources. Each predictor defined in text and in Table 1 footnotes. Predictors measured at the time of NDHS interview (household variables) or at cluster locations from the geospatial layers: rainfall and NDVI as year-on-year anomalies against the December 2021 to November 2022 baseline, conflict as log events over the December 2021 to May 2024 survey window, population density and travel time as static layers. |
| *If predictor measurement requires subjective interpretation, describe the qualifications and demographic characteristics of the predictor assessors* | **9c** | D;E | N/A. All predictors are objective measurements (counts, log values, geographic coordinates, satellite-derived indices). |
| SAMPLE SIZE *Explain how the study size was arrived at (separately for development and evaluation), and justify that the study size was sufficient to answer the research question. Include details of any sample size calculation* | **10** | D;E | Methods. Data sources. Sample size of 39,974 households is determined by the NDHS 2024 sampling design (1,380 EAs, ~30 households per EA). Power for tree-ensemble prediction on 15 features at this sample size is well above conventional rules of thumb (events-per-variable >> 100 with 27,508 outcome events). No formal a priori sample-size calculation. |
| MISSING DATA *Describe how missing data were handled. Provide reasons for omitting any data* | **11** | D;E | Methods. Data sources and analytical sample. 73 observations dropped for missing education of household head (otherwise complete-case). No imputation. |
| ANALYTICAL METHODS *Describe how the data were used (e.g., for development and evaluation of model performance) in the analysis, including whether the data were partitioned, considering any sample size requirements* | **12a** | D | Methods. Model specification, Evaluation. 80/20 cluster-grouped train-test split via GroupShuffleSplit on the enumeration-area identifier (31,975 train, 7,999 test households; 276 test EAs). Five-fold outcome-stratified, cluster-grouped cross-validation (StratifiedGroupKFold) on the training set for hyperparameter stability check. The household-level split is retained as a documented leakage comparison. |
| *Depending on the type of model, describe how predictors were handled in the analyses (functional form, rescaling, transformation, or any standardisation)* | **12b** | D | Methods. Predictor set, Model specification. Log transformations on count variables (ACLED events, population density). No scaling for tree models; logistic-regression baselines used same untransformed continuous variables. |
| *Specify the type of model, rationale, all model-building steps, including any hyperparameter tuning, and method for internal validation* | **12c** | D | Methods. Model specification. Four models compared: gradient-boosted trees (GBT) [4], random forest (RF) [5], single-level logistic regression, two-level mixed-effects logistic regression. Hyperparameters fixed a priori (GBT: 300 estimators, max depth 5, learning rate 0.1, subsample 0.8; RF: 200 trees, max depth 12, minimum leaf 5). Internal validation via the cluster-grouped held-out test set, grouped 5-fold CV, leave-zone-out spatial blocking, and a cluster bootstrap. |
| *Describe if and how any heterogeneity in estimates of model parameter values and model performance was handled and quantified across clusters (e.g., hospitals, countries). See TRIPOD-Cluster for additional considerations* | **12d** | D;E | Methods. Model specification, Evaluation. Two-level mixed-effects logistic regression with enumeration-area random intercepts; ICC reported (0.235 null, 0.206 full-covariate). Tree ensembles include cluster-level coordinates as predictors. |
| *Specify all measures and plots used (and their rationale) to evaluate model performance (e.g., discrimination, calibration, clinical utility) and, if relevant, to compare multiple models* | **12e** | D;E | Methods. Evaluation. AUROC with 1,000-iteration cluster-bootstrap 95 percent CI (enumeration areas resampled with replacement); Brier score; sensitivity, specificity, F1 at the training-frozen Youden-J threshold; decile calibration; leave-zone-out AUROC (Table 5); subgroup performance by zone, residence, and sex of head (Table 6); accumulated local effects as a PDP robustness check (S1 Fig). Reported in Tables 2, 5, 6 and Fig 3. |
| *Describe any model updating (e.g., recalibration) arising from the model evaluation, either overall or for particular sociodemographic groups or settings* | **12f** | E | N/A. No model updating performed. Calibration plot indicates post-hoc isotonic recalibration is not required for GBT in this application; Discussion notes recalibration as a standard pre-deployment safeguard. |
| *For model evaluation, describe how the model predictions were calculated (e.g., formula, code, object, application programming interface)* | **12g** | E | Methods. Model specification, Evaluation. SHAP values computed via the TreeExplainer algorithm in shap 0.43. All training, evaluation, and SHAP code deposited at https://doi.org/10.5281/zenodo.19738707 (this version: 10.5281/zenodo.21201585). |
| CLASS IMBALANCE *If class imbalance methods were used, state why and how this was done, and any subsequent methods to recalibrate the model or the model predictions* | **13** | D;E | Methods. Outcome variable, Model specification. Outcome prevalence 68.8 percent (moderate imbalance toward food-insecure). No resampling or class-weight adjustment used. No-skill Brier score (0.215) reported as benchmark; Brier skill scores reported relative to no-skill. |
| FAIRNESS *Describe any approaches that were used to address model fairness and their rationale* | **14** | D;E | Methods. Model interpretation, Discussion - Limitations. SHAP block decomposition contrasts household versus contextual contributions. Subgroup performance (AUROC, sensitivity, specificity, calibration-in-the-large) by geopolitical zone, urban-rural residence, and sex of household head is reported in Table 6 at the pooled training-frozen threshold; uneven zone-level error profiles are discussed with a recommendation for zone-specific thresholds. |
| MODEL OUTPUT *Specify the output of the prediction model (e.g., probabilities, classification). Provide details and rationale for any classification and how the thresholds were identified* | **15** | D | Methods. Model specification, Evaluation. Output: predicted probability of moderate-or-severe food insecurity. Binary classification at the Youden-J threshold determined on training set, fixed before test-set evaluation. |
| TRAINING vs EVAL *Identify any differences between the development and evaluation data in healthcare setting, eligibility criteria, outcome, and predictors* | **16** | D;E | Single dataset; no external validation. The cluster-grouped 80/20 split preserves identical setting, eligibility, outcome definition, and predictor set while keeping enumeration areas disjoint; leave-zone-out validation additionally quantifies performance under spatial extrapolation to unsampled zones (Table 5). |
| ETHICAL APPROVAL *Name the institutional research board or ethics committee that approved the study and describe the participant-informed consent or the ethics committee waiver of informed consent* | **17** | D;E | Methods. Ethics statement. Original 2024 NDHS protocol approved by the ICF Institutional Review Board (Rockville, Maryland, USA) and the National Health Research Ethics Committee of Nigeria. Informed consent obtained at NDHS interview by NPC and ICF following standard DHS Program protocol. No additional approval needed for secondary analysis of de-identified public-use microdata; data accessed 14 January 2026. |
| OPEN SCIENCE — FUNDING *Give the source of funding and the role of the funders for the present study* | **18a** | D;E | Submission portal Financial Disclosure (per PLOS policy, not in manuscript). 'The author received no specific funding for this work.' |
| CONFLICTS OF INTEREST *Declare any conflicts of interest and financial disclosures for all authors* | **18b** | D;E | Submission portal Competing Interests (per PLOS policy, not in manuscript). 'The author has declared that no competing interests exist.' |
| PROTOCOL *Indicate where the study protocol can be accessed or state that a protocol was not prepared* | **18c** | D;E | No prespecified study protocol was prepared. Hyperparameter values, train-test split, and Youden threshold were fixed before test-set evaluation, as documented in the deposited code at https://doi.org/10.5281/zenodo.19738707. |
| REGISTRATION *Provide registration information for the study, including register name and registration number, or state that the study was not registered* | **18d** | D;E | The study was not registered. Prediction-model studies are not subject to mandatory ICMJE clinical-trial registration. Reproducibility package and analysis code are publicly archived (Zenodo DOI above). |
| DATA SHARING *Provide details of the availability of the study data* | **18e** | D;E | Submission portal Data Availability statement. NDHS microdata available from the DHS Program at https://dhsprogram.com under standard data-use agreement; geospatial covariates available from CHIRPS (https://data.chc.ucsb.edu/products/CHIRPS-2.0/), MODIS MOD13A2 (https://lpdaac.usgs.gov and Google Earth Engine), ACLED (https://acleddata.com, free registration, event-level data not redistributable), WorldPop (https://www.worldpop.org), and the Nelson et al. 2019 travel-time surface (https://doi.org/10.6084/m9.figshare.7638134). |
| CODE SHARING *Provide details of the availability of the analytical code* | **18f** | D;E | All analysis code (household-split pipeline, cluster-grouped evaluation, Stata multilevel do-file, ALE and figure scripts, and the analytical-dataset derivation scripts), frozen result tables, and figures deposited at https://doi.org/10.5281/zenodo.19738707 (this version: 10.5281/zenodo.21201585) under MIT licence. Random seed fixed (seed = 243280004); package versions pinned. |
| PATIENT & PUBLIC INVOLVEMENT *Provide details of any patient and public involvement during the design, conduct, reporting, interpretation, or dissemination of the study or state no involvement* | **19** | D;E | No patient or public involvement. The study is a secondary analysis of public-use survey microdata; no participants were recruited or consulted by the author for design, conduct, interpretation, or dissemination. |
| RESULTS — PARTICIPANTS *Describe the flow of participants through the study, including the number of participants with and without the outcome and, if applicable, a summary of the follow-up time. A diagram may be helpful.* | **20a** | D;E | Methods. Data sources, Results → Sample characteristics, Table 1. NDHS 2024 sampled 40,047 households; 73 dropped for missing education of head; 39,974 retained. 27,508 (68.8 percent) classified food-insecure; 12,466 (31.2 percent) food-secure. |
| *Report the characteristics overall and, where applicable, for each data source or setting, including the key dates, key predictors (including demographics), treatments received, sample size, number of outcome events, follow-up time, and amount of missing data. A table may be helpful. Report any differences across key demographic groups.* | **20b** | D;E | Results. Sample characteristics, Table 1. Reports household composition, age and sex of head, education distribution, wealth quintiles, rural-urban share, geospatial covariates, and outcome by food-insecurity status. |
| *For model evaluation, show a comparison with the development data of the distribution of important predictors (demographics, predictors, and outcome).* | **20c** | E | N/A. Single dataset; train and test sets are drawn from the same population by stratified split, so by construction no distribution comparison is needed. |
| MODEL DEVELOPMENT *Specify the number of participants and outcome events in each analysis (e.g., for model development, hyperparameter tuning, model evaluation)* | **21** | D;E | Methods. Model specification, Evaluation; Results → Sample characteristics. Training set: 31,975 households (1,104 EAs); test set: 7,999 (276 EAs). Outcome events (food-insecure): ~22,000 in training, ~5,500 in test. Hyperparameters fixed before training; no separate tuning split. |
| MODEL SPECIFICATION *Provide details of the full prediction model (e.g., formula, code, object, application programming interface) to allow predictions in new individuals and to enable third-party evaluation and implementation, including any restrictions to access or re-use (e.g., freely available, proprietary)* | **22** | D | Full model specifications and prediction code deposited at https://doi.org/10.5281/zenodo.19738707 (this version: 10.5281/zenodo.21201585) under MIT licence. Repository includes 01_train_and_shap.py, 02_extended_evaluation.py, 03_figures.py, 04_multilevel_model.do, 05_grouped_evaluation.py, 06_ale_supporting_figure.py, 07_figures_grouped.py, and a derivation/ layer that rebuilds the analytical dataset from the raw sources with verification. |
| MODEL PERFORMANCE *Report model performance estimates with confidence intervals, including for any key subgroups (e.g., sociodemographic). Consider plots to aid presentation.* | **23a** | D;E | Results. Model performance, Table 2, Figure 3. AUROC with 1,000-iteration cluster-bootstrap 95 percent CIs on the grouped held-out set (RF 0.727 [0.704, 0.747]; GBT 0.714 [0.690, 0.737]; LR 0.679 [0.653, 0.702]). Brier scores, sensitivity, specificity, and F1 at the training-frozen threshold reported in Table 2; household-split results retained as a leakage comparison. Subgroup AUROC by zone, residence, and sex of head reported in Table 6; leave-zone-out AUROC in Table 5. |
| *If examined, report results of any heterogeneity in model performance across clusters. See TRIPOD Cluster for additional details.* | **23b** | D;E | Results. Model performance, Table 2. Multilevel logistic regression ICC reported (0.235 null, 0.206 full). Heterogeneity in predictive performance quantified across geopolitical zones by leave-zone-out validation (Table 5) and zone subgroup metrics (Table 6); the logistic baseline outperforms the ensembles in all six zones under spatial extrapolation. |
| MODEL UPDATING *Report the results from any model updating, including the updated model and subsequent performance* | **24** | E | N/A. No model updating performed. |
| INTERPRETATION *Give an overall interpretation of the main results, including issues of fairness in the context of the objectives and previous studies* | **25** | D;E | Discussion. Reassessing the household-block dominance, The travel-time paradox in the Nigerian context, Conflict and climate signals, Implications for applied food-insecurity prediction. Discusses the household-contextual decomposition as a range (42 to 52 percent contextual, above the 35 to 45 percent typical of earlier DHS tree-ensemble work but not a reversal), the travel-time association presented as a hypothesis for replication, conflict and climate signal sizes, the leave-zone-out reversal (linear baseline better under spatial extrapolation), and policy implications scoped to within-survey ranking. Zone-level fairness discussed with Table 6. |
| LIMITATIONS *Discuss any limitations of the study (such as a non-representative sample, sample size, overfitting, missing data) and their effects on any biases, statistical uncertainty, and generalizability* | **26** | D;E | Discussion. Limitations. Discusses cross-sectional design, single-country and single-survey-round generalisability, DHS GPS displacement as attenuating measurement error in fine-resolution covariates, the unweighted basis of the ML analysis, the sampling-frame exclusion of conflict-displaced households with an explicit scope statement, and the boundaries of generalizability beyond surveyed clusters as quantified by leave-zone-out validation. |
| USABILITY — DATA QUALITY *Describe how poor quality or unavailable input data (e.g., predictor values) should be assessed and handled when implementing the prediction model* | **27a** | D | Discussion. Limitations and Future work. Notes that the analytical pipeline assumes complete predictor coverage at the household level; missing-data handling for operational deployment requires extension. Model code includes basic input-validation checks. |
| USABILITY — USER INTERACTION *Specify whether users will be required to interact in the handling of the input data or use of the model, and what level of expertise is required of users* | **27b** | D | Discussion. Implications for applied food-insecurity prediction. Intended users are researchers and programme designers familiar with Python and statistical computing. Pipeline runs on a standard laptop in under 10 minutes. Documented in repository README. |
| FUTURE RESEARCH *Discuss any next steps for future research, with a specific view to applicability and generalizability of the model* | **27c** | D;E | Discussion. Future work. Lists temporal validation against 2021 Nigeria MICS-FIES data, replication in other DHS-FIES rounds, formal calibration audit on subgroups, and integration with Cadre Harmonisé / IPC area-level classifications. |
