# Supplementary material for: Household-level food-insecurity prediction from experience-scale surveys and open geospatial data: A reproducible machine-learning pipeline for Nigeria
Source: PLoS One. 2026 Oct 7;21(10):e0349978. doi: 10.1371/journal.pone.0349978 (PMC13645075; doi:10.1371/journal.pone.0349978)
Supplement: Supplementary file 2 [file pone.0349978.s004.docx]

Simulation notes — cluster-leakage magnitude under the PONE-D-26-22720 design

Purpose. The analytical CSV is not obtainable by the reviewer (DHS data-use agreement). This simulation generates data matching every design parameter the manuscript and README document, then runs the manuscript's evaluation design head-to-head against a cluster-grouped design using the manuscript's exact model specifications. It quantifies the leakage mechanism; it does not forecast the study's exact numbers.

Calibration (all from the manuscript/README). 39,974 households; 1,380 enumeration-area clusters (mean 29.0/cluster); outcome prevalence 0.688 (achieved exactly); cluster random effect sized for latent ICC 0.21 (empirical outcome ICC 0.249, vs. the study's null-specification 0.235); all eight contextual features constant within cluster; raw cluster coordinates carried as features; a genuine north–south outcome gradient carried by latitude; residual cluster structure (the manuscript's stated reason for retaining coordinates) independent across clusters; GBT 300/depth-5/lr-0.1/subsample-0.8, RF 200/depth-12/leaf-5, LR, seed 243280004 throughout. The simulated true signal is linear in standardized features, so by construction there is no real ensemble advantage in this world.

Headline results

| Design | Shared clusters | GBT | RF | LR | GBT no-coords |
| --- | --- | --- | --- | --- | --- |
| Household split (manuscript) | 1,378 / 1,380 | 0.840 | 0.833 | 0.817 | 0.826 |
| Cluster-grouped (corrected) | 0 | 0.792 | 0.801 | 0.810 | 0.726 |

SHAP coordinate share (GBT): 30.6% household-split vs 30.3% grouped. Household/contextual block split: 41.5/58.5 vs 43.2/56.8.

Bootstrap CI on identical grouped-split GBT predictions: iid [0.782, 0.803] (width 0.022) vs cluster bootstrap [0.773, 0.809] (width 0.037) — iid understates AUROC interval width ~1.7×.

Overfit gap: household GBT 0.072 (passes the manuscript's <0.15 trigger while leaking); grouped 0.121; grouped no-coords 0.180 (would fire the trigger).

What this confirms

1. The household-level split inflates tree-ensemble AUROC (GBT +4.8 points, RF +3.2) while leaving the linear baseline essentially unchanged (+0.7) — the leak operates through flexible learners memorizing cluster identity, exactly as the review states. The predicted near-total cluster overlap (1,378/1,380) was exact.
2. The design manufactures ensemble superiority from nothing. In a world with no true nonlinear advantage, the household split shows GBT beating LR by 2.3 points; the honest design shows LR ahead. The manuscript's 0.773-vs-0.681 margin is therefore unidentified under its own evaluation.
3. The manuscript's sole circuit-breaker is blind to its sole fatal flaw. The overfit-gap trigger passes the contaminated design comfortably and only begins to fire under honest evaluation — backwards as a safeguard.
4. The iid bootstrap materially understates uncertainty (~1.7× for AUROC under this design), so the paper's CI non-overlap argument requires a cluster bootstrap.

What this corrects in the reviewer's own claims

5. The "effective n ≈ 1,200" framing overstated the design effect for AUROC. DEFF ≈ 6.8 applies to prevalence-scale estimands; the measured AUROC CI-width inflation is ~1.7×. The review letter and instrument notes have been tempered accordingly. Direction unchanged; magnitude corrected.
6. Dropping the coordinates does not fix the leak. Household-split GBT *without* lat/long still scores 0.826 — ten points above its honest no-coordinate counterpart (0.726) — because the six remaining cluster-constant contextual features jointly fingerprint clusters. The split design, not the feature list, is the necessary fix. (This closes the most likely author-rebuttal path.)
7. Coordinates can carry genuine, generalizable signal, and SHAP shares do not diagnose leakage. In this simulation latitude carries a real regional gradient, so removing coordinates *hurts* honest out-of-cluster performance (0.792 → 0.726) and the SHAP block decomposition barely moves between designs. Consequence for the review: the manuscript's Table 4 decomposition is *unidentified* under the current design — it could collapse toward the household-dominant consensus or survive — and the grouped re-analysis is the only way to know. The review's demand is unchanged; the reviewer's directional prediction about Table 4 is withdrawn as unproven.

Limitations

Synthetic truth is linear and the latitude gradient is built in by construction; real data may contain genuine nonlinearities and a different gradient/noise split within the coordinate signal. All numbers here demonstrate the mechanism and its order of magnitude under the documented design parameters; none of them are predictions of the re-analyzed study's values.
