# Supplementary material for: Household-level food-insecurity prediction from experience-scale surveys and open geospatial data: A reproducible machine-learning pipeline for Nigeria
Source: PLoS One. 2026 Oct 7;21(10):e0349978. doi: 10.1371/journal.pone.0349978 (PMC13645075; doi:10.1371/journal.pone.0349978)
Supplement: Supplementary file 3 [file pone.0349978.s008.docx]

# Response to Reviewers (PONE-D-26-22720)

**Manuscript:** Household-level food-insecurity prediction from experience-scale surveys and open geospatial data: a reproducible machine-learning pipeline for Nigeria

**Author:** Philip Osung

**Date:** 5 July 2026

Dear Dr. Jahun,

Thank you for the careful editorial assessment. We thank both reviewers for the very thorough reviews. Reviewer 2 in particular conducted a code-level audit, supplied a remediation script and simulation evidence, and identified a genuine flaw in our evaluation design. We have re-analysed the data under the corrected design, restated every affected claim from the new results, and strengthened the reproducibility deposit. The paper’s conclusions have changed where the evidence required it, and we believe the manuscript is materially better for it.

A note on how we implemented the corrected evaluation. Rather than running the script Reviewer 2 generously supplied, we wrote an independent implementation of the corrected design (cluster-grouped holdout, grouped cross-validation, cluster bootstrap, coordinate ablation, spatially blocked validation, and subgroup evaluation), froze its results, and only then ran the reviewer’s script unmodified on an isolated copy of the data. The two implementations agree to machine precision on every shared deterministic quantity (holdout AUROC, Brier scores, thresholds, sensitivity and specificity, all cross-validation folds, the SHAP block decomposition, leave-zone-out results, and subgroup metrics), and bootstrap intervals agree within Monte Carlo noise. Our implementation (05_grouped_evaluation.py) is included in the updated deposit; the reviewer’s script is their own work, so rather than redistributing it we document the validation run and its agreement here.

Point-by-point responses follow. Reviewer and editor text is abridged in places for readability; no point has been omitted. Line references are to the clean revised manuscript.

## Academic Editor comments

**AE-1. “Occasionally overstating findings, particularly claims about ‘revising the empirical consensus’ and policy applicability. Please soften the tone.”**

Done, and the corrected analysis itself enforced this. The abstract, introduction, discussion, and conclusions no longer claim to revise the consensus. Under the corrected cluster-grouped evaluation, the household-versus-contextual SHAP decomposition spans 47.7 to 57.6 percent household share depending on the model and on whether raw coordinates are included. The revised claim is a range: contextual predictors carry between 42 and 52 percent of predictive signal, larger than the 35 to 45 percent typical of earlier DHS tree-ensemble work, but not a reversal of it (abstract, lines 16 to 18; introduction, lines 68 to 73). Policy-applicability language has been scoped to within-survey household ranking, with a new explicit limitation for displaced populations (see R2-2 below) and a new finding that for extrapolation to unsampled areas the logistic baseline outperformed the ensembles in all six geopolitical zones.

**AE-2. “Travel-time paradox seems to be speculative; the data is insufficient to support this proposal.”**

We agree, and the revised manuscript presents the subsistence-buffer interpretation strictly as a hypothesis for replication. The empirical association itself is design-respecting and survives the corrected evaluation: the multilevel model with enumeration-area random intercepts estimates OR 0.790 (95 percent CI 0.734 to 0.849) for travel time, and travel time ranks third in the grouped-evaluation SHAP at 8.7 percent. What changed is the interpretive register: the mechanism language (“protectively associated,” “subsistence-buffer effect”) has been replaced by “associated with lower food-insecurity risk,” the measurement-error alternatives are given equal standing, and the manuscript states plainly that no mechanism can be established from one cross-section (travel-time subsection, lines 376 to 401).

**AE-3. “Limited discussion of potential biases, survey weighting, external validation, and generalizability.”**

The revised Methods adds a paragraph on survey weights (lines 211 to 216) (the ML task estimates unweighted household-level risk ranking; design-weighted prevalence figures are cited from the Final Report and flagged as such; the multilevel benchmark respects the design through enumeration-area random intercepts). The revised Results adds two forms of design-respecting validation: leave-zone-out spatial blocking (new Table 5, lines 348 to 357) and subgroup performance by zone, residence, and sex of household head (new Table 6, lines 361 to 374). The Limitations section now covers the DHS GPS displacement effect on the geospatial covariates, the sampling-frame exclusion of displaced households with an explicit scope statement, the unweighted basis of the ML analysis, and the boundaries of generalizability outside surveyed clusters, which the leave-zone-out results quantify directly (lines 521 to 542).

## Journal requirements

**JR-1. PLOS ONE style and file naming.** The revised submission follows the PLOS ONE file-naming conventions: Manuscript, Revised Manuscript with Track Changes, Response to Reviewers, Fig1 to Fig4 (300 dpi TIFF within the size limits), S1_Fig.tif, S1_Table.csv, and the updated TRIPOD-AI checklist.

**JR-2. Code sharing.** The reproducibility deposit has been extended to contain all author-generated code underpinning the findings: the original pipeline scripts, the new cluster-grouped evaluation script (05_grouped_evaluation.py), the Stata do-file for the multilevel models (previously absent, as Reviewer 2 noted), and the analytical-dataset derivation code. A new version of the Zenodo deposit has been published under the record’s concept DOI (10.5281/zenodo.19738707, which always resolves to the latest version); this version is 10.5281/zenodo.21201585. The deposit’s frozen “expected outputs” have been updated to the corrected results.

**JR-3. New Figure 4.** All four figures have been regenerated from the corrected (cluster-grouped) models at 300 dpi within the PLOS dimension limits, Figure 2 has been recomputed on the probability scale (see the corrections section below), and Figure 4 has been rebuilt for legibility.

**JR-4. Supporting Information captions.** Captions for both Supporting Information files (S1 Fig, S1 Table) appear at the end of the manuscript (lines 649 to 658), and both are cited in the text (lines 156 and 393).

**JR-5. Citation recommendations.** Neither reviewer recommended specific citations. No citations were added on that basis.

## Reviewer 1

**R1-1. Validation strategy: household-level random split permits cluster and spatial leakage; repeat the evaluation using cluster-disjoint validation, preferably with spatially blocked or leave-region-out validation, and report AUROC, calibration, Brier score, and SHAP/block-decomposition results under these stricter settings.**

We have done exactly this, and the reviewer’s concern was borne out. The entire evaluation was repeated with enumeration-area-grouped splits (GroupShuffleSplit on the DHS cluster identifier for the 80/20 holdout; StratifiedGroupKFold for cross-validation), so that no enumeration area contributes households to both training and test sets (Methods, lines 157 to 172). Under the corrected design, on a held-out test set of 276 enumeration areas (7,999 households):

- GBT AUROC falls from 0.773 to 0.714 (cluster-bootstrap 95 percent CI 0.690 to 0.737); RF from 0.763 to 0.727 (0.704 to 0.747); the logistic baseline is essentially unchanged at 0.679 (0.653 to 0.702). The pattern (tree ensembles inflated, linear model untouched) is the signature of the cluster-memorisation mechanism the reviewer described.
- Brier scores under the grouped design: 0.188 (GBT), 0.185 (RF), 0.195 (LR). Calibration-in-the-large is within 0.003 of zero for all three models.
- Leave-zone-out validation (training on five geopolitical zones, scoring the sixth) is reported in new Table 5 (lines 348 to 357). The logistic baseline outperforms GBT in all six zones (mean AUROC 0.671 versus 0.628). The revised Discussion states plainly that the ensemble advantage does not survive spatial extrapolation.
- The SHAP block decomposition was re-estimated on the grouped models and is reported with cluster-bootstrap confidence intervals (new Table 4; see R1-2).

All headline numbers in the abstract, results, discussion, and conclusions have been restated from the corrected analysis. The Methods now explains why household-level splits leak on two-stage cluster samples, and the manuscript presents the household-split results alongside the grouped results as a quantified demonstration of the leakage mechanism.

**R1-2. Treat SHAP-share claims more cautiously; mean absolute SHAP shares can be unstable with correlated predictors; include household-only, contextual-only, combined-without-coordinates, and grouped permutation-importance models, ideally with uncertainty intervals for the SHAP block shares.**

All requested specifications are now reported, with uncertainty intervals.

- Block decomposition with cluster-bootstrap 95 percent intervals (1,000 enumeration-area resamples): household share 47.7 percent (46.4 to 49.1) for GBT and 51.3 percent (49.7 to 53.2) for RF with the full feature set; 51.9 percent (50.6 to 53.2) and 57.6 percent (56.3 to 59.0) respectively without raw coordinates.
- Feature-set ablations (new rows in Table 2): without coordinates, GBT 0.692, RF 0.700, LR 0.662; household-only, GBT 0.625, RF 0.638, LR 0.625; contextual-only, GBT 0.635, RF 0.647, LR 0.568; all with cluster-bootstrap intervals.
- Joint block-permutation importance as a SHAP-free check: permuting the household block jointly drops grouped-split GBT AUROC by 0.168 against 0.121 for the contextual block (full set), and 0.164 against 0.094 without coordinates.

The revised text reports the contextual share as a range across these specifications (42 to 52 percent) rather than a point claim, and notes that SHAP and permutation attribution give bounds on the answer from opposite directions.

**R1-3. Travel-time interpretation too strong for a cross-sectional predictive study; present as hypothesis; test whether the association persists without coordinates or with stronger geographic controls; consider ALE/ICE or subgroup analyses.**

The interpretation has been reframed as a hypothesis throughout (see AE-2). On persistence: travel time remains the third-ranked SHAP feature (8.7 percent) under the grouped design with coordinates present, retains its negative direction in the coordinate-ablated models, and the design-respecting multilevel estimate (OR 0.790, 95 percent CI 0.734 to 0.849), which conditions on zone effects and enumeration-area random intercepts, is unchanged. Following the reviewer’s suggestion we additionally computed accumulated local effects (ALE), which avoid the extrapolation into unpopulated feature-space regions that partial dependence permits with correlated predictors. The ALE curve confirms the direction and adds resolution: predicted risk is elevated for households within roughly one hour of a city (ALE +0.048 at zero hours), crosses zero near 1.1 hours, and is reduced across the more remote deciles (-0.056 at the most remote decile), a pattern consistent with the market-exposure reading of the subsistence-buffer hypothesis. ALE panels for the four highest-SHAP features appear as new supporting figure S1 (cited at line 393; caption at lines 650 to 655), with the computation script included in the deposit.

**R1-4. Additional issues: survey design and weights; GPS displacement justification; multilevel benchmark held-out performance not reported; overextended consensus claims; cropped or hard-to-read figures.**

Weights: addressed in a new Methods paragraph (see AE-3). GPS displacement: the Methods now states the displacement magnitude (2 to 10 km), why the 50 km conflict buffer and kilometre-scale climate rasters are tolerant of them, and that fine-resolution covariates (travel time, population density) carry attenuating measurement error as a result; the Limitations reiterate this. Multilevel benchmark: the revised text clarifies its role as an inferential benchmark estimated on the full sample, and the out-of-sample discrimination comparison is carried by the single-level logistic regression fit on the identical grouped split; we now state explicitly that the multilevel model’s held-out discrimination is not reported because its purpose is variance decomposition and design-respecting effect estimation, not prediction. Consensus claims: withdrawn and restated as a range (see AE-1). Figures: all four regenerated at 300 dpi from the corrected models.

## Reviewer 2

We are grateful to Reviewer 2 for the depth of this review: a full code audit, a design-calibrated simulation, a corrected-evaluation script, and a readiness scorecard. The paper is substantially stronger for it.

**R2-Major-1. The train/test split leaks cluster information, inflating all reported performance; re-evaluate with cluster-grouped splits and spatially blocked validation; re-run SHAP and Table 4 on grouped models; remove or re-justify raw coordinates; restate all headline claims; re-merge hv001 or derive cluster membership from coordinates.**

Accepted in full. The corrected analysis is described under R1-1 and R1-2 above; we summarise the reviewer-specific points here.

- Cluster identifier: the analytical dataset already carries hv001 as cluster_id (1,380 enumeration areas; the unique coordinate pairs map one-to-one to it, as the reviewer’s fallback anticipated). The deposit’s documented schema omitted this column; the documentation has been corrected and the derivation code now states it explicitly.
- The reviewer’s prediction that the leak operates through flexible learners was exact: the ensembles lose 4 to 6 AUROC points under the grouped design; the linear baseline moves by 0.2 points. The reviewer’s simulation estimated inflation of 4.8 (GBT) and 3.2 (RF) points; the actual values are 5.9 and 3.7.
- Coordinates: retained in the primary specification under the corrected design, now with an explicit justification (they carry a genuine north-south gradient; the grouped split prevents their use as cluster fingerprints), and the full coordinate-ablation results are reported alongside (Table 2, Table 4). We note the reviewer’s simulation finding that removing coordinates under a household-level split would not have fixed the leak; our ablation results under the grouped design are consistent with that analysis.
- Table 4 and all SHAP results are re-estimated on grouped models with cluster-bootstrap intervals. The decomposition shifted from 45.9/54.1 (GBT, household/contextual) to 47.7/52.3 with coordinates and 51.9/48.1 without; RF shifts to 51.3/48.7 and 57.6/42.4. The “consensus revision” claim is withdrawn in favour of the range statement (AE-1).
- Every headline claim has been restated from the corrected numbers. The household-split results remain in the manuscript only as a quantified comparison demonstrating the leakage mechanism.

Verification: our independent implementation and the reviewer’s supplied script, run unmodified on an isolated copy, produce identical results for every shared deterministic quantity (maximum difference 7e-15 on the block decomposition; bootstrap intervals within 0.002, attributable to RNG stream ordering). We report this agreement so the re-analysis can be checked without repeating it.

**R2-Major-2. No subgroup performance on an outcome whose prevalence ranges from 37.9 to 94.1 percent across states; report sensitivity, specificity, calibration at the deployed threshold by zone, urban/rural, sex of household head, on the corrected split; add an explicit scope statement for displaced populations.**

Done. New Table 6 reports, at the training-frozen Youden threshold on the grouped holdout: AUROC from 0.674 (North Central) to 0.756 (South East); sensitivity falling to 0.622 in the North West, where false negatives concentrate; specificity falling to 0.413 in the North East, where prevalence is highest and false positives concentrate; calibration-in-the-large within plus-or-minus 0.031 across all ten subgroups. The Results text draws the operational conclusion the reviewer pointed to: a pooled threshold misallocates differently by zone, and zone-specific thresholds should be expected in any deployment.

The scope statement is added to the Limitations: the model should not be applied to conflict-displaced populations in camps or informal settlements without separate validation, because the DHS sampling frame excludes them and no performance evidence exists for that population. The Introduction’s targeting paragraph now carries a forward reference to this limit.

**R2-Major-3. Statistical inference ignores the sampling design: iid bootstrap understates AUROC interval width; use a cluster bootstrap; reconcile the “stratified bootstrap” footnote and the “validated by five-fold cross-validation” wording with the code.**

Accepted. All AUROC confidence intervals are now from a 1,000-iteration cluster bootstrap that resamples enumeration areas with replacement, retaining their households (Methods, lines 188 to 195). As the reviewer’s simulation anticipated, intervals widened materially (GBT full-set width 0.047 under the cluster bootstrap against 0.022 under the iid bootstrap of the original submission). The original CI non-overlap argument does not survive: the GBT and LR intervals overlap under the corrected design, and the manuscript no longer makes that claim. The RF and LR intervals are marginally disjoint, and we state this cautiously without resting any headline on it.

Text-code reconciliations: the Table 2 footnote now describes the cluster bootstrap accurately (the “stratified bootstrap” wording is gone), and the Methods states that hyperparameters were fixed a priori at conventional defaults and not tuned, with grouped cross-validation reported as a stability check rather than as selection.

**R2-Moderate-1. The Data Availability Statement claims more than the deposit contains: the Stata multilevel code is absent; the analytical-dataset derivation exists only as README prose; the schema lacks the EA identifier; “publicly available without restriction” is inaccurate for ACLED.**

All four points are corrected.

1. The Stata do-file estimating the multilevel models (ICCs in Table 2; the odds ratios cited in the Discussion, including travel time) is now in the deposit.
2. The analytical-dataset derivation (DHS recode merges, the five geospatial joins, anomaly baselines, FIES outcome construction, the 50 km conflict buffer) is now deposited as code rather than prose, with the merge order and parameters explicit (deposit derivation/ folder, three scripts with a built-in verification mode).
3. The documented CSV schema now includes the enumeration-area identifier (hv001 as cluster_id), which the corrected evaluation requires.
4. The Data Availability Statement now states per-source access terms; for ACLED it notes free registration and redistribution restrictions.

The deposit’s README “expected outputs” section has been updated so that reproducers verify against the corrected numbers, per the reviewer’s minor comment. A new Zenodo version has been published (10.5281/zenodo.21201585).

**R2-Minor-1. Add assertion checks or frequency printouts for all recoded indicators; the female_head recode follows the same pattern as the corrected urban_rural_gps bug and is unverified.**

Done. The evaluation script now asserts plausibility bounds on every recoded indicator at load time (female-head prevalence within 0.10 to 0.40; rural within 0.30 to 0.70; cluster count within 1,300 to 1,450; coordinate-pair groups equal to the cluster count) and fails loudly on violation. For the record, the female_head recode verifies correctly on the analytical dataset (21.1 percent female-headed).

**R2-Minor-2. Abstract: “travel time … is protectively associated with food insecurity” should read “associated with lower food-insecurity risk.”**

Done, in the abstract and at every occurrence in the body text.

**R2-Minor-3. Update the README frozen results after re-analysis so verification targets corrected values.**

Done as part of the deposit update: the do-file is scripts/04_multilevel_model.do in the new deposit version (10.5281/zenodo.21201585).

**R2-Minor-4. ACLED access wording.**

Done (see R2-Moderate-1, point 4).

## Corrections identified by the authors during this revision

While preparing the derivation code for deposit (Reviewer 2, moderate concern 1), we audited the full provenance of the five geospatial covariates against the code that originally constructed the analytical dataset. The audit identified three description errors in the submitted Methods. None affects any computed value in the paper; every number is computed from the analytical dataset, which is unchanged. All three descriptions are corrected in the revised manuscript, and the deposited derivation code documents the true definitions with verification against the analytical dataset.

1. **Rainfall and NDVI baselines.** The submitted Methods described anomalies relative to long-run climatologies (1981 to 2020 for CHIRPS; 2001 to 2020 for MODIS). The constructed variables in fact compare the survey year against the immediately preceding twelve months (December 2021 to November 2022), with the rainfall survey year being January to December 2023 and the NDVI survey year December 2022 to November 2023 (MOD13A2 at 1 km, not MOD13Q1 at 250 m). The variables are therefore year-on-year anomalies. We note this is arguably the more relevant shock measure for the 2023 to 2024 macroeconomic context the paper studies; the Methods (lines 107 to 121), Table 1 footnotes (lines 229 to 241), and product citation have been corrected accordingly.
2. **Conflict window.** The submitted text described ACLED counts over the 12 months preceding interview. The constructed variable counts events across the full December 2021 to May 2024 survey window. Corrected in the Methods (line 115) and the Table 1 footnote (line 233).
3. **Travel-time layer.** The submitted text described travel time to the nearest city of at least 50,000 population from the Malaria Atlas Project surface. The constructed variable is travel time to the nearest city of one to five million inhabitants, from the Nelson et al. (2019) global accessibility suite, which is computed on the Malaria Atlas Project friction surface of Weiss et al. (2018). The Nelson reference has been added (reference 30; appended at the end of the list to avoid wholesale renumbering of the Vancouver sequence in tracked changes, and we are happy to renumber at proof) and every mention corrected. Substantively this sharpens the finding: the travel-time association concerns remoteness from major metropolitan markets, which is consistent with the market-exposure reading discussed in the paper.

These errors were found because the reviewer’s insistence on deposited derivation code forced a complete provenance audit. We consider this the review process working as intended, and we have added assertion-backed verification to the deposit so that the documented definitions and the dataset cannot drift apart again.

Separately, our own pre-submission check caught a labelling error in Figure 2 of the original submission: the partial-dependence panels were computed by scikit-learn’s recursion method, which returns values on the model’s decision-function (log-odds) scale, while the axis label claimed the probability scale, and the caption misdescribed partial dependence as holding other features at their means. Figure 2 has been recomputed by direct averaging of predicted probabilities, so the axis now shows what it claims; the caption has been corrected (lines 381 to 385); and the associated wealth-gradient sentence in the Discussion (line 379) now reads a reduction of roughly 0.61 across the observed range of the wealth index (previously “approximately 0.35”, a figure inherited from the mislabelled scale). Feature rankings, model fits, and all reported performance metrics are unaffected.

## Closing

The corrected evaluation changed this paper’s headline numbers, weakened one claim to the point of withdrawal, softened a second into a range, and surfaced a new finding (the leave-zone-out reversal) that we believe is of independent interest to the applied literature. We are grateful to both reviewers for the rigour that produced this outcome, and we hope the revised manuscript now meets PLOS ONE’s publication criteria.

Sincerely,

Philip Osung Department of Economics,

Nile University of Nigeria

ORCID 0009-0001-6700-372X
