# Supplementary material for: Household-level food-insecurity prediction from experience-scale surveys and open geospatial data: A reproducible machine-learning pipeline for Nigeria
Source: PLoS One. 2026 Oct 7;21(10):e0349978. doi: 10.1371/journal.pone.0349978 (PMC13645075; doi:10.1371/journal.pone.0349978)
Supplement: Supplementary file 4 [file pone.0349978.s009.docx]

# Response to Reviewers (PONE-D-26-22720R1)

**Manuscript:** Household-level food-insecurity prediction from experience-scale surveys and open geospatial data: a reproducible machine-learning pipeline for Nigeria

**Author:** Philip Osung

**Date:** 10 September 2026

Dear Dr. Jahun,

We thank both reviewers for the second reading. All four points raised in this round are addressed below. The changes are wording-level; no analysis, figure, or deposited result has changed, and the reproducibility deposit (10.5281/zenodo.21201585) remains the version reviewed. Line references are to the clean revised manuscript.

## Reviewer 1

**R1-1. Survey-design terminology: “respects the sampling design” could be read as covering weights and stratification, not only clustering.**

Agreed, and reworded as suggested. The sentence now reads: “The multilevel benchmark accounts for the clustering induced by the two-stage sample through its enumeration-area random intercepts; sampling weights and stratification are not incorporated in that model” (lines 215 to 217), which limits the claim to what the model does.

**R1-2. GPS displacement: avoid implying that displacement necessarily attenuates estimated associations.**

Agreed. The Limitations sentence now states that the displacement and the city-size band “may attenuate or otherwise distort estimated coefficients on the spatial variables” (lines 537 to 538), replacing the earlier wording that asserted attenuation as the expected direction.

**R1-3. Travel-time heterogeneity across wealth and residence subgroups.**

We agree this is the right next test of the hypothesis and have added the statement the reviewer suggested at the end of the travel-time results: “Whether the association varies across wealth and residence subgroups is not tested here; wealth-stratified and residence-stratified estimation is a priority for future validation” (lines 405 to 406). We prefer to leave the stratified estimation to future work rather than add a post hoc analysis in this round, consistent with the pre-specified scope of the corrected evaluation.

## Reviewer 2

**R2-1. The grouped GBT train-test gap (0.160) slightly exceeds the paper’s own 0.15 re-specification trigger; a one-line acknowledgment that the random-forest and range-based conclusions carry the weight would suffice.**

Added. The Results now state, immediately after the gap figures: “Consistent with the pre-specified rule, the conclusions of this paper rest on the random forest and on ranges across models rather than on the gradient-boosted model alone” (lines 263 to 265). We thank the reviewer for holding us to our own rule.

**R2-2. Deployment-readiness items (subgroup accessibility of targeting actions, staged implementation pathway, stopping rules), raised as non-blocking and forward-looking.**

We acknowledge these as the right checklist for any move from research use to program targeting. They sit outside the paper’s stated within-survey scope, as the reviewer notes, and the manuscript’s scope statement and displaced-population caveat already mark that boundary. We will carry these items into any future operationalisation work.

We also thank the reviewer for the independent three-decimal validation of the manuscript’s numerical claims against the deposit and for confirming the corrected source provenance against the archives.

## Journal requirements

**Citation recommendations.** Neither reviewer recommended specific citations; no citations were added on that basis.

**Reference list.** We checked all 30 references against retraction notices and publisher records. None is retracted and none carries an expression of concern. The check surfaced one citation error carried over from the original submission: reference 26 was miscited. It has been corrected to Key, Sadoulet and de Janvry (2000), Transactions costs and agricultural household supply response, American Journal of Agricultural Economics, 82(2), 245 to 259, which is the work the in-text claim about transaction costs and market access rests on. This is the only change to the reference list, made as a tracked change.

Sincerely,

Philip Osung

Nile University of Nigeria

ORCID 0009-0001-6700-372X
