# Supplementary material for: Household-level food-insecurity prediction from experience-scale surveys and open geospatial data: A reproducible machine-learning pipeline for Nigeria
Source: PLoS One. 2026 Oct 7;21(10):e0349978. doi: 10.1371/journal.pone.0349978 (PMC13645075; doi:10.1371/journal.pone.0349978)
Supplement: Peer review history

# Peer review: Decision Letter 0

**DOI:** 10.1371/journal.pone.0349978.r001

**Authors:** Ibrahim Jahun

8 Jun 2026

Dear Dr. Osung,

**Academic Editor Comments:**

This paper presents a machine-learning model for predicting household food insecurity in Nigeria using the 2024 NDHS Food Insecurity Experience Scale (FIES) combined with open geospatial dataset. The topic is timely, policy relevant, and align well with current interest about food security, geospatial analytics, and transparent machine learning.

The manuscript however requires improvement to strengthen the findings and usability. Here are some observations for the authors to address in addition to comments provided by the two reviewers below.

- Occasionally overstating findings, particularly claims about "revising the empirical consensus" and policy applicability. Please soften the tone.
- Travel-time paradox (greater remoteness associated with lower food insecurity) seems to be speculative; the data is insufficient to support this proposal.
- Limited discussion of potential biases, survey weighting, external validation, and generalizability.

==============================

Please submit your revised manuscript by Aug 07 2026 11:59PM. If you will need more time than this to complete your revisions, please reply to this message or contact the journal office at plosone@plos.org. When you're ready to submit your revision, log on to https://www.editorialmanager.com/pone/ and select the 'Submissions Needing Revision' folder to locate your manuscript file.

Please include the following items when submitting your revised manuscript:

- A letter that responds to each point raised by the academic editor and reviewer(s). You should upload this letter as a separate file labeled 'Response to Reviewers'.
- A marked-up copy of your manuscript that highlights changes made to the original version. You should upload this as a separate file labeled 'Revised Manuscript with Track Changes'.
- An unmarked version of your revised paper without tracked changes. You should upload this as a separate file labeled 'Manuscript'.

If you would like to make changes to your financial disclosure, please include your updated statement in your cover letter. Guidelines for resubmitting your figure files are available below the reviewer comments at the end of this letter.

As the corresponding author, your ORCID iD is verified in the submission system and will appear in the published article. PLOS supports the use of ORCID, and we encourage all coauthors to register for an ORCID iD and use it as well. Please encourage your coauthors to verify their ORCID iD within the submission system before final acceptance, as unverified ORCID iDs will not appear in the published article. *Only*  the individual author can complete the verification step; PLOS staff *cannot*  verify ORCID iDs on behalf of authors.

We look forward to receiving your revised manuscript.

Kind regards,

Ibrahim Jahun, MD, MSC, PhD

Academic Editor

PLOS One

**Journal Requirements:**

1. When submitting your revision, we need you to address these additional requirements.

Please ensure that your manuscript meets PLOS ONE's style requirements, including those for file naming. The PLOS ONE style templates can be found at

https://journals.plos.org/plosone/s/file?id=wjVg/PLOSOne_formatting_sample_main_body.pdf and

https://journals.plos.org/plosone/s/file?id=ba62/PLOSOne_formatting_sample_title_authors_affiliations.pdf

2. Please note that PLOS One has specific guidelines on code sharing for submissions in which author-generated code underpins the findings in the manuscript. In these cases, we expect all author-generated code to be made available without restrictions upon publication of the work. Please review our guidelines at https://journals.plos.org/plosone/s/materials-and-software-sharing#loc-sharing-code and ensure that your code is shared in a way that follows best practice and facilitates reproducibility and reuse.

3. Please upload a new copy of Figure 4, as the detail is not clear. Please follow the link for more information:  https://journals.plos.org/plosone/s/figures

4. Please include captions for your Supporting Information files at the end of your manuscript, and update any in-text citations to match accordingly. Please see our Supporting Information guidelines for more information: http://journals.plos.org/plosone/s/supporting-information.

5. If the reviewer comments include a recommendation to cite specific previously published works, please review and evaluate these publications to determine whether they are relevant and should be cited. There is no requirement to cite these works unless the editor has indicated otherwise.

[Note: HTML markup is below. Please do not edit.]

Reviewers' comments:

Reviewer's Responses to Questions

**Comments to the Author**

1. Is the manuscript technically sound, and do the data support the conclusions?

Reviewer #1: Partly

Reviewer #2: Partly

**********

2. Has the statistical analysis been performed appropriately and rigorously?

Reviewer #1: No

Reviewer #2: Yes

**********

3. Have the authors made all data underlying the findings in their manuscript fully available?

The PLOS Data policy

Reviewer #1: Yes

Reviewer #2: Yes

**********

4. Is the manuscript presented in an intelligible fashion and written in standard English?

Reviewer #1: Yes

Reviewer #2: Yes

**********

**Reviewer #1:**  This study addresses an important and policy-relevant problem by developing a reproducible machine-learning pipeline for predicting household-level moderate-or-severe food insecurity in Nigeria using the 2024 NDHS FIES module and open geospatial covariates. However, in its current form, the central empirical claims are not yet sufficiently supported for publication.

A major concern relates to the validation strategy. The reported 80/20 household-level random split may allow substantial cluster and spatial leakage because multiple households from the same enumeration areas, sharing cluster-level coordinates and contextual covariates, are likely to appear in both training and test sets. This issue is particularly consequential because latitude, longitude, and geospatial predictors are included in the model. As a result, the headline AUROC, SHAP decomposition, and policy interpretation may overstate true out-of-cluster predictive performance. I strongly recommend that the authors repeat the evaluation using cluster-disjoint validation, preferably with additional spatially blocked or leave-state/leave-region-out validation, and report AUROC, calibration, Brier score, and SHAP/block-decomposition results under these stricter settings.

Claims about the near-equal contribution of household and contextual predictors should also be treated more cautiously. Mean absolute SHAP shares can be unstable in the presence of correlated predictors and may partly reflect residual spatial structure captured by latitude and longitude rather than interpretable contextual mechanisms. Robustness checks should include household-only, contextual-only, combined-without-coordinates, and grouped permutation-importance models, ideally with uncertainty intervals for the SHAP block shares.

The interpretation of travel time to the nearest city as a protective “subsistence-buffer” effect is interesting but currently too strong for a cross-sectional predictive study. It would be more appropriate to present this as a hypothesis rather than a confirmed mechanism. Additional tests should examine whether the association persists after removing coordinates or adding stronger geographic controls, and the authors should consider ALE/ICE plots or subgroup analyses by rural status and wealth.

Several additional issues also require attention: limited treatment of the NDHS complex survey design and sampling weights, insufficient justification of the effect of DHS GPS displacement on fine-resolution geospatial covariates, incomplete comparability of the multilevel logistic benchmark because held-out predictive performance is not reported, and overextended claims about revising the empirical consensus that appear insufficiently supported by the cited literature. Some figures also appear visually cropped or difficult to read and should be reformatted.

This is a promising manuscript with a relevant research question, a valuable dataset, and a potentially useful reproducible pipeline. Nevertheless, substantial methodological robustness analyses and more cautious interpretation are required before the reported conclusions can be considered reliable enough for publication.

**Reviewer #2:**  Reviewer Summary:

This manuscript combines the 2024 NDHS FIES module with five open geospatial layers to predict moderate-or-severe household food insecurity, and deposits a seeded, open-licensed pipeline.

The reproducibility practices, the multilevel regression cross-check, and the candor of the Limitations section are well above the norm for this literature, and I commend the author for them. This allowed for near perfect reproduction, aside from the portions stated below.

The submission of the code also allowed for complete code review and identification of code flaws and suggestion for remediation. The authors practice and code submission is an excellent example of how ML manuscripts should be submitted to allow reviewers to actually review the methodology, statistics and deployment readiness and to suggest genuinely constructive corrective actions to the authors.

The manuscript, data and code were all evaluated against a published AI readiness for clinical practice framework which generated a reviewer generated Python script which measures six readiness evaluations. The json output has been included for the authors to review.

The manuscript has many strengths; however, the evaluation design contains a structural flaw — cluster-level information leaking across the train/test split — that invalidates the headline performance numbers and the paper's central empirical claim as currently reported. The flaw is fully fixable with the data and code already in hand, and I encourage resubmission after the re-analysis described below.

Recommendation: Major revision.

Major concerns

1. The train/test split leaks cluster information, inflating all reported performance.

The NDHS is a two-stage cluster sample: the analytical dataset contains 39,974 households drawn from 1,380 enumeration areas, roughly 29 households per cluster. All eight contextual predictors — including raw cluster latitude and longitude — are constant within a cluster. The 80/20 split is performed at the household level, so with near-certainty every cluster contributes households to both the training and test sets.

Under these conditions, a depth-5 boosted ensemble can isolate individual clusters through the coordinate pair and learn each cluster's training-set outcome prevalence; test households from the same clusters are then predicted by recall rather than generalization. The manuscript itself supplies the evidence on both sides: the Methods state that coordinates were retained "so that the learner could absorb residual spatial structure not carried by the five geospatial covariates" (lines 127–128), which under a household-level split is precisely the leakage mechanism; and the multilevel model reports an intraclass correlation of 0.206 after covariates (line 205), quantifying the within-cluster dependence the ML evaluation assumes away. Consistent with memorization, latitude and longitude together carry 19.9% of total SHAP (Table 3, ranks 2 and 4). The overfit-gap diagnostic (line 169) cannot detect this failure mode, because cluster memorization generalizes perfectly to held-out households from the same clusters.

Three downstream claims are contaminated: (a) the test-set AUROC of 0.773; (b) the ensemble-over-logistic margin, since a linear model cannot exploit coordinates the way trees can, so the leak manufactures exactly the advantage the abstract reports; and (c) the household-versus-contextual decomposition, the paper's flagship result.

As a first-order check, removing the two coordinate features from the contextual block and renormalizing yields approximately 57/43 in favor of the household block — inside the 55–65% consensus range the paper claims to revise. Whether the coordinate share reflects cluster memorization or a genuine, generalizable regional gradient is precisely what the grouped re-analysis will determine; under the current design the decomposition is unidentified in either direction.

Corrective recommendations:

re-evaluate with cluster-grouped splits (e.g., GroupShuffleSplit / GroupKFold keyed on EA identifier) for both the holdout and the five-fold cross-validation

report spatially blocked validation (e.g., leave-state-out) as a stricter robustness check, since the stated use case — targeting in areas the survey never sampled — is generalization to new locations; re-run the SHAP analysis and Table 4 on the grouped models; and either remove raw coordinates from the feature set or re-justify their inclusion under the corrected design. (removing the coordinates while keeping the household-level split will not remediate the error, because the remaining six cluster-constant contextual features still jointly fingerprint clusters; the split design, not the feature list, is the necessary fix.)

All headline claims should then be restated from the uncontaminated numbers. (the documented analytical-dataset schema contains no EA identifier, so either re-merge the cluster number (hv001) from the household recode, or — equivalently for this dataset — derive cluster membership by grouping on the unique (latitude, longitude) pair, which is constant within each enumeration area and should yield exactly 1,380 groups)

2. No subgroup performance is reported, on an outcome whose prevalence ranges from 37.9% to 94.1% across states.

All performance metrics are pooled. For a model proposed as an alternative targeting instrument for the National Social Safety Nets Project and state cash-transfer schemes, pooled metrics can conceal exactly where false negatives concentrate — a missed food-insecure household in conflict-affected Yobe is not interchangeable with one in Lagos. Please report sensitivity, specificity, and calibration at the deployed threshold, stratified at minimum by geopolitical zone, urban/rural residence, and sex of household head, computed on the corrected (cluster-grouped) split.

Relatedly, the Limitations section correctly notes that conflict-displaced households in camps and informal settlements fall outside the DHS sampling frame. Given the targeting framing, this deserves more than a limitation: please add an explicit scope statement that the model should not be applied to displaced populations without separate validation.

3. Statistical inference ignores the sampling design.

The 1,000-iteration bootstrap resamples households as if independent. With ~29 correlated households per cluster and an ICC of 0.206, the iid bootstrap materially understates interval width for AUROC — in a simulation calibrated to this study's documented design parameters (sample size, cluster count, prevalence, ICC), cluster-bootstrap AUROC intervals are roughly 1.7 times wider than iid intervals computed on identical predictions — so the CI non-overlap argument (lines 200–202) requires re-testing under a cluster bootstrap. (For prevalence-scale estimands the design effect is larger still, approximately 1 + (m−1)·ICC ≈ 6.8.) Please use a cluster bootstrap (resample enumeration areas, retaining their households). Two related text–code reconciliations: Table 2 footnote b describes a "stratified bootstrap," but the deposited code performs an unstratified resample that skips degenerate draws; and the Methods describe the fixed hyperparameters as "validated by five-fold stratified cross-validation," when the CV evaluates pre-chosen defaults rather than performing any selection — please state how the values were chosen.

Moderate concern:

1. The Data Availability Statement claims more than the deposit contains. The statement that "all analysis code... required to reproduce the results reported in this paper are deposited" is not currently accurate on two counts.

First, the multilevel logistic regression — which produces the ICC estimates in Table 2 and the odds ratios cited in the Discussion, including the travel-time result — was fit in Stata outside the deposited Python pipeline, and no Stata code appears in the package. As it stands, the deposited code reproduces the machine-learning results affected by Major concern 1, while the multilevel results — arguably the paper's most defensible findings — have no deposited code at all. Please add the Stata do-file.

Second, the construction of the analytical dataset exists only as a README walkthrough, which states that "the merge proceeds in the obvious order"; for the anomaly baselines, the 50 km conflict buffer, and the FIES outcome derivation, the order and parameters are not obvious, and small choices in these steps move the results. Please deposit the derivation code as well. (The DHS access restriction itself is appropriately handled and consistent with journal policy; these points concern code, not data.) The pinned environment, fixed seed, and frozen outputs are commendable. Note also that the documented CSV schema contains no enumeration-area identifier, which is required for the corrected evaluation under Major concern 1.

Minor concerns:

The code comments disclose a corrected bug in which a string-typed column silently produced an all-zero rural indicator, and the README documents the trap in its sanity-check section. Documentation is not enforcement: please add assertion checks or frequency printouts in the code for all recoded indicators (the female_head recode follows the identical pattern and is currently unverified).

Abstract: "travel time to the nearest city is protectively associated with food insecurity" should read "associated with lower food-insecurity risk."

The README's "What you should see" section freezes the current (contaminated) numbers as the correctness oracle for reproducers. After the re-analysis, please update the frozen results, figures, and expected-output section so that verification targets the corrected values.

The Data Availability Statement describes the geospatial covariates as "publicly available without restriction," but ACLED requires registration (as the README correctly notes) and its terms of use restrict redistribution. Please adjust the wording.

Irrespective of re-analysis all of the following findings hold:

The multilevel logistic results — including the travel-time association (OR 0.790, 95% CI 0.734–0.849) and the null NDVI effect — are estimated with enumeration-area random intercepts appropriate to the design and survive independently of the concerns above.

The ICC estimates, the dominance of the wealth index, the documentation of data sources, and the open-release ethos are genuine contributions.

The travel-time paradox in particular remains an interesting finding; after the corrected evaluation it may well be the paper's strongest empirical result.

suggestions for code remediation are attached and 04_group_evaluation.py and the final review script json file is attached for author review

**********

what does this mean?). If published, this will include your full peer review and any attached files.

If you choose “no”, your identity will remain anonymous but your review may still be made public.

**Do you want your identity to be public for this peer review?**  For information about this choice, including consent withdrawal, please see our Privacy Policy

Reviewer #1: No

Reviewer #2: **Yes:**  A.C Demidont, DO

**********

[NOTE: If reviewer comments were submitted as an attachment file, they will be attached to this email and accessible via the submission site. Please log into your account, locate the manuscript record, and check for the action link "View Attachments". If this link does not appear, there are no attachment files.]

To ensure your figures meet our technical requirements, please review our figure guidelines: https://journals.plos.org/plosone/s/figures

You may also use PLOS’s free figure tool, NAAS, to help you prepare publication quality figures: https://journals.plos.org/plosone/s/figures#loc-tools-for-figure-preparation.

NAAS will assess whether your figures meet our technical requirements by comparing each figure against our figure specifications.

Attachment Submitted filename: demidont_simulation_notes.docx

Attachment Submitted filename: 04_grouped_evaluation.py

Attachment Submitted filename: answers_22720_corrected.json

# Author comment: Author response to Decision Letter 1

**DOI:** 10.1371/journal.pone.0349978.r002

18 Jul 2026

A complete point-by-point response to the Academic Editor, Journal Requirements, and both reviewers is provided in the attached "Response to Reviewers" file, with line references to the clean revised manuscript. In brief: the entire evaluation was repeated under cluster-grouped data splits as required by both reviewers; all affected results, claims, figures, and the reproducibility deposit (10.5281/zenodo.19738707; this version 10.5281/zenodo.21201585) have been updated accordingly; and four description errors identified during the authors' own checks are corrected and disclosed in the response letter.

Attachment Submitted filename: Response to Reviewers.docx

# Peer review: Decision Letter 1

**DOI:** 10.1371/journal.pone.0349978.r003

**Authors:** Ibrahim Jahun

8 Sep 2026

Dear Dr. Osung,

Thank you for submitting your manuscript to PLOS One. After careful consideration, we feel that it has merit but does not fully meet PLOS One’s publication criteria as it currently stands. Therefore, we invite you to submit a revised version of the manuscript that addresses the points raised during the review process.

**ACADEMIC EDITOR:**

Please submit your revised manuscript by Oct 08 2026 11:59PM. If you will need more time than this to complete your revisions, please reply to this message or contact the journal office at plosone@plos.org. When you're ready to submit your revision, log on to https://www.editorialmanager.com/pone/ and select the 'Submissions Needing Revision' folder to locate your manuscript file.

- A letter that responds to each point raised by the academic editor and reviewer(s). You should upload this letter as a separate file labeled 'Response to Reviewers'.
- A marked-up copy of your manuscript that highlights changes made to the original version. You should upload this as a separate file labeled 'Revised Manuscript with Track Changes'.
- An unmarked version of your revised paper without tracked changes. You should upload this as a separate file labeled 'Manuscript'.

If you would like to make changes to your financial disclosure, please include your updated statement in your cover letter. Guidelines for resubmitting your figure files are available below the reviewer comments at the end of this letter.

We look forward to receiving your revised manuscript.

Kind regards,

Ibrahim Jahun, MD, MSC, PhD

Academic Editor

PLOS One

Journal Requirements:

1. If the reviewer comments include a recommendation to cite specific previously published works, please review and evaluate these publications to determine whether they are relevant and should be cited. There is no requirement to cite these works unless the editor has indicated otherwise.

2. Please review your reference list to ensure that it is complete and correct. If you have cited papers that have been retracted, please include the rationale for doing so in the manuscript text, or remove these references and replace them with relevant current references. Any changes to the reference list should be mentioned in the rebuttal letter that accompanies your revised manuscript. If you need to cite a retracted article, indicate the article’s retracted status in the References list and also include a citation and full reference for the retraction notice.

Reviewers' comments:

Reviewer's Responses to Questions

**Comments to the Author**

Reviewer #1: (No Response)

Reviewer #2: All comments have been addressed

**********

2. Is the manuscript technically sound, and do the data support the conclusions?

Reviewer #1: Yes

Reviewer #2: Yes

**********

3. Has the statistical analysis been performed appropriately and rigorously?

Reviewer #1: Yes

Reviewer #2: Yes

**********

4. Have the authors made all data underlying the findings in their manuscript fully available?

The PLOS Data policy

Reviewer #1: Yes

Reviewer #2: Yes

**********

5. Is the manuscript presented in an intelligible fashion and written in standard English?

Reviewer #1: Yes

Reviewer #2: Yes

**********

Reviewer #1: The authors have responded carefully and substantively to the concerns raised in the previous round. In particular, the revised validation strategy, the additional robustness analyses for the SHAP-based decomposition, and the more cautious interpretation of the travel-time finding have considerably strengthened the manuscript. I believe that the major concerns have been satisfactorily addressed.

I have only a few minor suggestions that may further improve the methodological precision and clarity of the manuscript before publication:

1. Survey-design terminology. I suggest slightly clarifying the statement that the multilevel model “respects the sampling design” through enumeration-area random intercepts. Random intercepts appropriately account for clustering at the enumeration-area level, but this wording could potentially be interpreted as encompassing all elements of the complex survey design, including unequal selection probabilities, sampling weights, and stratification. A more narrowly phrased statement distinguishing clustering from the broader survey-design features would improve precision.

2. Interpretation of GPS displacement. The discussion of DHS coordinate displacement and its implications for fine-resolution geospatial covariates is useful. I suggest using cautious wording when describing the expected direction of the resulting measurement error. Rather than implying that displacement necessarily attenuates estimated associations, it may be more appropriate to state that it can introduce measurement error and potentially attenuate or otherwise distort spatial associations, depending on the underlying spatial structure and covariate.

3. Travel-time robustness and heterogeneity. The additional coordinate-ablation, multilevel, and ALE analyses provide useful support for the robustness of the observed travel-time association. As a further clarification, the authors may wish to comment briefly on whether this association could vary across socioeconomic or rural-urban subgroups, particularly by household wealth. If such an analysis is beyond the intended scope of the present revision, a short statement identifying this as an avenue for future validation would be sufficient.

These points are intended primarily as clarifications rather than requests for substantial additional analysis. Overall, the manuscript has improved considerably, and I would support publication after these minor issues are addressed.

Reviewer #2: I commend the authors for an exceptionally thorough and good-faith revision. The evaluation has been rebuilt on cluster-grouped (enumeration-area) splits with a cluster bootstrap, spatially-blocked leave-zone-out validation, and subgroup performance reporting; the reproducibility deposit is now complete (the multilevel do-file and the analytical-dataset derivation code were added); and every headline claim has been restated from the corrected analysis — including withdrawing the "consensus revision" claim in favor of a calibrated range. The authors also independently reimplemented the corrected-evaluation design and report agreement with the reviewer-supplied script to machine precision. The deposit is a genuinely runnable artifact (pinned environment, fixed seeds, derivation scripts, and frozen result tables), and I validated every numerical claim in the revised manuscript to three decimal places against it; the one script that regenerates the headline results from the restricted DHS microdata (05_grouped_evaluation.py) cannot be re-executed by a third party because that microdata is not redistributable, so for those values I verified against the deposited frozen outputs rather than a fresh run. I also confirmed the external data provenance: the corrected NDVI (MODIS MOD13A2), rainfall (CHIRPS), and travel-time (Nelson/Weiss accessibility surface) source descriptions each check out against their source archives. Every substantive concern from the first round is resolved, and the paper is materially stronger for it.

One minor, non-blocking note: under the corrected grouped design the full-feature gradient-boosted model's train–test AUROC gap (~0.16) slightly exceeds the paper's own 0.15 re-specification trigger, so the random-forest and range-based conclusions are the ones to rely on; a one-line acknowledgment would suffice and is not a barrier to acceptance.

For completeness and transparency, my structured readiness script continues to flag three deployment-readiness items — accessibility of targeting actions across subgroups, a staged implementation pathway, and explicit stopping rules/circuit-breakers. These fall outside the paper's stated within-survey research scope and are non-blocking; I raise them only as forward-looking considerations should the pipeline later be operationalized for program targeting.

**********

what does this mean?). If published, this will include your full peer review and any attached files.

If you choose “no”, your identity will remain anonymous but your review may still be made public.

**Do you want your identity to be public for this peer review?**  For information about this choice, including consent withdrawal, please see our Privacy Policy

Reviewer #1: No

Reviewer #2: No

**********

[NOTE: If reviewer comments were submitted as an attachment file, they will be attached to this email and accessible via the submission site. Please log into your account, locate the manuscript record, and check for the action link "View Attachments". If this link does not appear, there are no attachment files.]

To ensure your figures meet our technical requirements, please review our figure guidelines: https://journals.plos.org/plosone/s/figures

You may also use PLOS’s free figure tool, NAAS, to help you prepare publication quality figures: https://journals.plos.org/plosone/s/figures#loc-tools-for-figure-preparation.

NAAS will assess whether your figures meet our technical requirements by comparing each figure against our figure specifications.

# Author comment: Author response to Decision Letter 2

**DOI:** 10.1371/journal.pone.0349978.r004

11 Sep 2026

A point-by-point response to the Academic Editor and both reviewers is provided in the attached "Response to Reviewers" file, with line references to the clean revised manuscript. In brief: the survey-design statement now claims only the clustering handled by the enumeration-area random intercepts (lines 215 to 217); the GPS-displacement wording no longer implies attenuation is the necessary direction of measurement error (lines 537 to 538); the travel-time discussion flags wealth-stratified and residence-stratified estimation as a priority for future validation (lines 405 to 406); and the Results state that conclusions rest on the random forest and on ranges across models, consistent with the paper's pre-specified 0.15 overfitting trigger (lines 263 to 265). A retraction check of all 30 references found none retracted; one miscited entry (reference 26) was corrected to Key, Sadoulet and de Janvry (2000) as a tracked change and is disclosed in the response letter. No analysis, result, figure, or deposited file has changed.

Attachment Submitted filename: Response_to_Reviewers_auresp_2.docx

# Peer review: Decision Letter 2

**DOI:** 10.1371/journal.pone.0349978.r005

**Authors:** Ibrahim Jahun

23 Sep 2026

Household-level food-insecurity prediction from experience-scale surveys and open geospatial data: a reproducible machine-learning pipeline for Nigeria

PONE-D-26-22720R2

Dear Dr. Osung,

We’re pleased to inform you that your manuscript has been judged scientifically suitable for publication and will be formally accepted for publication once it meets all outstanding technical requirements.

Within one week, you’ll receive an e-mail detailing the required amendments. When these have been addressed, you’ll receive a formal acceptance letter and your manuscript will be scheduled for publication.

An invoice will be generated when your article is formally accepted. Please note, if your institution has a publishing partnership with PLOS and your article meets the relevant criteria, all or part of your publication costs will be covered. Please make sure your user information is up-to-date by logging into Editorial Manager at Editorial Manager® and clicking the ‘Update My Information' link at the top of the page. For questions related to billing, please contact billing support.

If your institution or institutions have a press office, please notify them about your upcoming paper to help maximize its impact. If they’ll be preparing press materials, please inform our press team as soon as possible -- no later than 48 hours after receiving the formal acceptance. Your manuscript will remain under strict press embargo until 2 pm Eastern Time on the date of publication. For more information, please contact onepress@plos.org.

Kind regards,

Ibrahim Jahun, MD, MSC, PhD

Academic Editor

PLOS One

Additional Editor Comments (optional):

Reviewers' comments:

Reviewer's Responses to Questions

**Comments to the Author**

Reviewer #1: All comments have been addressed

**********

2. Is the manuscript technically sound, and do the data support the conclusions?

Reviewer #1: Yes

**********

3. Has the statistical analysis been performed appropriately and rigorously?

Reviewer #1: Yes

**********

4. Have the authors made all data underlying the findings in their manuscript fully available?

The PLOS Data policy

Reviewer #1: Yes

**********

5. Is the manuscript presented in an intelligible fashion and written in standard English?

Reviewer #1: Yes

**********

Reviewer #1: The authors have satisfactorily addressed the comments raised in the previous round of review. The revisions regarding survey-design terminology, GPS displacement, and the interpretation and future validation of the travel-time association adequately resolve my remaining concerns. I have no further substantive comments and consider the manuscript suitable for publication.

**********

what does this mean?). If published, this will include your full peer review and any attached files.

If you choose “no”, your identity will remain anonymous but your review may still be made public.

**Do you want your identity to be public for this peer review?**  For information about this choice, including consent withdrawal, please see our Privacy Policy

Reviewer #1: No

**********

# Editor report: Acceptance letter

**DOI:** 10.1371/journal.pone.0349978.r006

**Authors:** Ibrahim Jahun

PONE-D-26-22720R2

PLOS One

Dear Dr. Osung,

I'm pleased to inform you that your manuscript has been deemed suitable for publication in PLOS One. Congratulations! Your manuscript is now being handed over to our production team.

At this stage, our production department will prepare your paper for publication. This includes ensuring the following:

* All references, tables, and figures are properly cited

* All relevant supporting information is included in the manuscript submission,

* There are no issues that prevent the paper from being properly typeset

You will receive further instructions from the production team, including instructions on how to review your proof when it is ready. Please keep in mind that we are working through a large volume of accepted articles, so please give us a few days to review your paper and let you know the next and final steps.

Lastly, if your institution or institutions have a press office, please let them know about your upcoming paper now to help maximize its impact. If they'll be preparing press materials, please inform our press team within the next 48 hours. Your manuscript will remain under strict press embargo until 2 pm Eastern Time on the date of publication. For more information, please contact onepress@plos.org.

You will receive an invoice from PLOS for your publication fee after your manuscript has reached the completed accept phase. If you receive an email requesting payment before acceptance or for any other service, this may be a phishing scheme. Learn how to identify phishing emails and protect your accounts at https://explore.plos.org/phishing.

If we can help with anything else, please email us at customercare@plos.org.

Thank you for submitting your work to PLOS One and supporting open access.

Kind regards,

PLOS One Editorial Office Staff

on behalf of

Dr. Ibrahim Jahun

Academic Editor

PLOS One
